# A Swedish dietary guideline index, gut microbial α-diversity and prevalence of metabolic syndrome – observations in the Swedish CArdioPulmonary bioImage Study (SCAPIS)

**DOI:** 10.29219/fnr.v68.10547

**Published:** 2024-11-28

**Authors:** Ulrika Ericson, Sophie Hellstrand, Anna Larsson, Mariam Miari, Sergi Sayols-Baixeras, Koen F Dekkers, Göran Bergström, Andrei Malinovschi, Gunnar Engström, Johan Ärnlöv, Tove Fall, Marju Orho-Melander

**Affiliations:** 1 Department of Clinical Sciences in Malmö, Diabetes and Cardiovascular Disease, Lund University, Malmö, Sweden; 2 Molecular Epidemiology, Department of Medical Sciences, Uppsala University, Uppsala, Sweden; 3 SciLifeLab, Uppsala University, Uppsala, Sweden; 4 CIBER Cardiovascular Diseases (CIBERCV), Instituto de Salud Carlos III, Madrid, Spain; 5 Department of Molecular and Clinical Medicine, Institute of Medicine, Sahlgrenska Academy, University of Gothenburg, Gothenburg, Sweden; 6 Department of Clinical Physiology, Sahlgrenska University Hospital, Region Västra Götaland, Gothenburg, Sweden; 7 Department of Medical Sciences, Clinical Physiology, Uppsala University, Uppsala, Sweden; 8 Department of Clinical Sciences in Malmö, Cardiovascular Research-Epidemiology, Lund University, Malmö, Sweden; 9 Division of Family Medicine and Primary Care, Department of Neurobiology, Care Science and Society, Karolinska Institutet, Huddinge, Sweden; 10 School of Health and Social Studies, Dalarna University, Falun, Sweden

**Keywords:** food patterns, epidemiology, metabolic syndrome, gut microbiota

## Abstract

**Background:**

Metabolic syndrome (MetS) is characterized by coexisting risk factors for type 2 diabetes and cardiovascular disease. Diet is of importance in their aetiology, and gut microbiota (GM) may constitute a link between diet and metabolic health. Understanding the interplay between diet and GM could contribute novel insights for future dietary guidelines, and aid in preventive actions to motivate adherence to dietary guidelines.

**Objective:**

We intended to create a Swedish dietary guideline index (SweDGI) measuring adherence to 12 Swedish dietary guidelines and examine whether SweDGI and its components are associated with GM α-diversity (Shannon index) and prevalent MetS, and if the association between the Shannon index and MetS differs depending on SweDGI.

**Design:**

SweDGI was based on food-frequency data assessed 2014–2018 in 10,396 diabetes-free participants from the Malmö and Uppsala-sites of the Swedish CArdioPulmonary bioImage Study (SCAPIS) (50–64 y, 53% women). We estimated the Shannon index from shotgun metagenomic sequencing-data to assess microbial richness and evenness. We used a general linear model to examine cross-sectional SweDGI-Shannon associations and logistic regression for associations with MetS.

**Results:**

Most guidelines were followed by less than half of the participants. Men showed poorer adherence. Higher SweDGI was linked to higher Shannon index (*P*-trend across five SweDGI-groups = 1.7 × 10^-12^). Most guidelines contributed to this observation. Higher SweDGI and Shannon index were associated with lower MetS-prevalence, where the lowest prevalence was observed among those with both high SweDGI and high Shannon index (odds ratio:0.43; 95% confidence interval:0.35, 0.52). Both the Shannon index and SweDGI were associated with MetS, independently of the level of the other factor (*P*-interaction = 0.82).

**Conclusions:**

We created a new index to comprehensively reflect adherence to the Swedish dietary guidelines in sub-cohorts within the large multicentre SCAPIS study. Better adherence was associated with a richer and more even GM and lower prevalence of MetS. The inverse association between the Shannon index and MetS was consistent at different levels of adherence to dietary guidelines.

## Popular scientific summary

- An index was created to assess overall adherence to 12 Swedish dietary guidelines in 10,396 adults.
- Higher adherence to the guidelines was linked to a richer and more even gut microbiota.
- The lowest prevalence of metabolic syndrome was seen in the group of participants with both high adherence to dietary guidelines and a rich, balanced gut microbiota.
- The relevance of our findings needs evaluation in studies with incident cases of type 2 diabetes and cardiovascular disease.

Food-based dietary guidelines (FBDGs), which are very similar worldwide, are considered to have an important role in national health efforts as poor diet contributes to a large part of the enormous burden of non-communicable diseases, including type 2 diabetes and cardiovascular disease (1, 2). Prevalence of metabolic syndrome (MetS) is characterized by clustering of risk factors for type 2 diabetes and cardiovascular disease with high prevalence globally (3, 4). Diet is a complex exposure of interacting foods and nutrients, making it crucial to examine both adherence to overall healthy food patterns and specific dietary intakes in relation to metabolic health.

Dietary factors including intakes of specific foods, such as fruit and vegetables, or macro- and micronutrients could affect the development of MetS via different mechanisms such as effects on body fat or circulating levels of triglycerides and glucose (5–9). Diet is also an important determinant of the gut microbiota (GM) composition and richness (10), which have been found to be altered in many disease states including obesity, type 2 diabetes and cardiovascular disease (11–14). Several specific dietary components have been suggested to alter the GM, which may causally affect the development of type 2 diabetes by producing short-chain fatty acids and other bioactive compounds from fermentable dietary fibres, proteins and polyphenols (15, 16). Dietary factors may also influence circulating levels of other GM-derived compounds of importance to metabolic health, such as bile acids or trimethylamine N-oxide (17, 18). Although important interaction between a range of dietary factors and GM has been acknowledged, a deeper understanding of this phenomenon is missing, and knowledge about such interplay has only to a very small extent contributed to the evidence basis for current dietary guidelines (16, 19).

We intended to create a Swedish dietary guideline index (SweDGI) to assess overall adherence to national FBDGs (20), as well as adherence to each guideline, in two sub-cohorts from the large Swedish CArdioPulmonary bioImage Study (SCAPIS). By using the Shannon index to reflect α-diversity, we then examined if SCAPIS participants adhering to the dietary guidelines had a more diverse GM and if they were less likely to have MetS. We also examined whether gut microbial α-diversity was associated with MetS. Finally, we hypothesized that the association between gut microbial α-diversity and MetS may differ depending on SweDGI, as we believe that by providing essential substrates, a healthy dietary pattern could be a prerequisite for optimal metabolic benefits from a favourable GM.

## Methods

### Study population and data collection

SCAPIS is a population-based cohort including 30,154 women and men, invited at age 50–64 to cardiovascular and pulmonary examinations conducted in 2014–2018 in six university hospitals in Sweden (21). Deep shotgun metagenomic sequencing was performed to characterize the GM of SCAPIS participants from the Malmö and Uppsala sites. In total, 11,287 participants came from Malmö (*n* = 6,251) and Uppsala (*n* = 5,036). The participation rate was 53% in Malmö and 47% in Uppsala. After excluding participants without dietary data (*n* = 36), 11,251 participants remained. Similarly to most epidemiological studies, we used cutoffs to define plausible energy intake (22) and excluded participants who reported extreme energy intakes (women: < 500 or > 5,000 kcal/day, men: < 550 or > 6,000 kcal/day) (*n* = 149). We then also excluded participants with known diabetes at baseline (based on questionnaire data or medical interview at baseline) (*n* = 582) and/or unknown prevalence of MetS (*n* = 148), due to missing data on the waist (*n* = 4), systolic blood pressure (*n* = 90), diastolic blood pressure (*n* = 92), high-density lipoprotein (HDL) cholesterol (*n* = 48), triglycerides (*n* = 47) and/or glucose (*n* = 14). Our study sample then consisted of 10,396 individuals (Supplementary Fig. 1). Among those, metagenomic GM data were missing in 1,295 individuals (1,285 participants did not provide faecal samples, mainly due to that the faecal sampling in Malmö was initiated later than the main data collection, and metagenomic sequencing data were missing in 10 participants). Therefore the study sample for analyses involving GM α-diversity was reduced to 9,101 individuals. As the use of antibiotics is known to alter the GM composition, we performed sensitivity analysis on GM in a further reduced sample (*n* = 8,109) after the exclusion of individuals who used antibiotics during the 6 months preceding data collection (*n* = 992).

The study protocols were in accordance with the Declaration of Helsinki and were approved by the regional ethics committee (Umeå: Dnr 2010-228-31M; Uppsala: Dnr 2018/315). All participants provided written informed consent.

### Dietary data

Diet was assessed with the web-based food frequency questionnaire MiniMeal-Q (23). It is a self-administered, semi-quantitative questionnaire including questions about meal patterns, and frequencies and portion sizes of single foods and mixed dishes covering a period of the past few months. Because of its dynamic structure, it includes between 75 and 126 food items. Most questions have an optional answering frequency in a nine-grade scale from ‘five times per day’ to ‘one-to-three times per month’. The food frequency questionnaire data were linked to the Swedish food composition database (Livsmedelsdatabasen, version 2012-01-06) to calculate the average daily intake of nutrients. Estimation of portion sizes to calculate the average daily intake of foods in the current study was based on weights per standard units (24), weights per standard portions (25) and photo-options presented to the participants in SCAPIS: (1) meat, chicken, fish and vegetarian substitutes; (2) potatoes, rice and pasta; and (3) vegetables (both raw and cooked). The relative validity of MiniMeal-Q has been evaluated by comparing the reported nutrient and energy intakes with that of weighed 7-day food records and objectively measured total energy expenditure (TEE) using the doubly labelled water technique (23, 26, 27). Intakes of fruits, vegetables and whole grains have been validated against dietary plasma biomarkers (28).

#### Description of SweDGI and the included index components

SweDGI was created to capture the study participants’ overall level of adherence to 12 national FBDGs proposed by the National Food Agency in 2015 (20) while considering the possibilities and limitations of data obtained from MiniMeal-Q (Supplementary Tables 1 and 2). We could, for example, take advantage of the fact that MiniMeal-Q included a question about salting at the table. However, since it did not provide information on the most commonly used cooking fats and fat spreads, we only included the intake of oil dressings as a marker for the consumption of healthy food fats. The SweDGI reflects consumption of the following seven encouraged intake components: (1) fruits; (2) vegetables; (3) legumes; (4) nuts and seeds; (5) whole grains; (6) oil-dressing and (7) fish and shellfish, and the following five components for foods to restrict: (1) red and processed meat; (2) high-fat dairy products; (3) added sugar; (4) alcohol and (5) salt. Added sugar intake was obtained by summing the participants’ intakes of sucrose and monosaccharides, followed by subtraction of the estimated intakes of those sugars from commonly consumed fruits and vegetables in Sweden [(sucrose intake + fructose intake + glucose intake) – (fruit and berry intake × 0.10) – (vegetable intake × 0.03) – (juice intake × 0.08)].

As described in Supplementary Tables 1 and 2, for each of the components included in the SweDGI, individuals were assigned a score between 0 and 4 points to estimate the degree of adherence, where 4 was defined as best adherence to a guideline. With the exception of salt, points were awarded based on the following scheme: 4 points (very good adherence to the dietary guideline) = intake in line with the recommended intake level; 3 points (good adherence) = intake < 25% from the recommended level; 2 points (moderate adherence) = intake 25–50% from the recommended level; 1 point (poor adherence) = intake 50–75% from the recommended level; 0 points (very poor adherence) = intake > 75% from the recommended value. Regarding encouraged foods, falling short of the dietary recommendations implied that the individual consumed *less* than the recommended value. On the other hand, for foods to be restricted, failing to meet the dietary guidelines implied that the individual consumed *more* than the recommended value. The salt component was based on two criteria: (1) the first criterion reflected intake of salt from all foods consumed except salt added at the table and instead of 0–4 points it gave 0–3 points depending on the level of adherence to the salt guideline, and (2) the second criterion reflected salting at the table; adherence to this criterion gave 0–1 points and 1 point was distributed to those who did not report that they used to add salt at the table. A 5-level scale was used for the components because previous studies have suggested that indices comprised of multifaceted components capture variations in the GM composition more effectively than indices composed of binary variables that only indicate adherence or non-adherence to the dietary guidelines (29). To overcome the issue of measuring absolute intakes and minimize the effects of misreporting, we additionally examined a version of the SweDGI in which all components were expressed as intakes per MJ (instead of absolute intakes (g/d) in line with how most of the guidelines are stated), as described in detail in Supplementary Table 2.

The points were aggregated into a single cumulative score; the minimum score an individual could receive was zero while the maximum was 48. The participants were then categorized into five groups based on their cumulative score, indicating their overall level of adherence to the Swedish dietary guidelines: (1) ≤ 18 points; (2) 19–22 points; (3) 23–25 points; (4) 26–29 points and (5) ≥30 points, using the binning function in SPSS to make as equal groups as possible.

### Clinical measurements

Body weight was measured on a balance scale with subjects dressed in light clothing without shoes. Height and waist were measured according to current recommendations (30). Systolic and diastolic blood pressures were measured twice in each arm with an automatic device (Omron M10-IT, Omron Health Care Co, Kyoto, Japan) and the mean of the measurements was used. Venous blood samples (100 mL) were collected from the participants after an overnight fast and used immediately for standard laboratory analyses including HDL cholesterol, triglycerides and plasma glucose. Body mass index (BMI; in kg/m^2^) was calculated.

### Other variables

Data on age and sex were obtained from the Swedish Population Register. Information on smoking (never, ex- or current smoker), leisure time physical activity (sedentary, moderate, regular moderate or intensive), education (incomplete primary school, primary school, upper secondary or university degree), medication and whether the participants were born in Sweden (yes or no) was collected with a standardized questionnaire (21).

### Use of antibiotics

Prescription data from the Swedish drug registry were used to identify individuals using antibiotics (ATC code J01) during the 6 months preceding the data collection at baseline.

### Definition of the MetS

In total 3,222 study participants (31% of all participants) were classified as having MetS. The classification was based on international consensus criteria that the International Diabetes Federation and the American Heart Association/National Heart, Lung, and Blood Institute have agreed to (4) with cut points for blood pressure (systolic ≥ 130 mm Hg and or diastolic ≥ 85), fasting glucose (≥ 5.6 mmol/L), triglycerides (≥ 1.7 mmol/L), HDL cholesterol (≤ 1.29 mmol/L for women and ≤ 1.03 mmol/L for men) and waist circumference (≥ 94 cm for men and ≥ 80 cm for women). Self-reported treatments with antihypertensive or lipid-lowering drugs were also used as criteria for hypertension and dyslipidaemia. In line with the international consensus, prevalent MetS was defined as having at least 3 out of the 5 criteria (4).

### Gut microbiota

DNA extraction from faecal samples, metagenomic sequencing and taxonomic profiling were performed at Clinical Microbiomics A/S (Copenhagen, Denmark) as earlier described (31). In brief, libraries of fragmented DNA were sequenced with the Illumina Novaseq 6,000 platform. Nonhost reads and reads from public metagenomes were used to build separate nonredundant gene catalogues. Signature gene sets of metagenomic species were defined as bins of co-abundant genes (32). Species abundances were estimated by mapping reads to the signature gene sets followed by normalization by effective gene lengths. To make it relative, the normalized counts were divided by the total counts of each sample and then multiplied by 100. Downsized relative abundance was estimated by random sampling without replacement from the gene count table corresponding to the signature genes. After removing an outlier sample, data were downsized to 210,430 reads. To measure the participants’ overall species richness and evenness, α-diversity was assessed with the Shannon diversity index using the R v4.1.1 package *vegan* v2.5–7 using downsized data (33).

### Statistical analysis

The SPSS statistical computer package (version 29.0; IBM Corporation, Armonk, NY, USA) was used for all statistical analyses.

We used the general linear model to examine continuous baseline characteristics across categories of the SweDGI, across quintiles of the Shannon index and in cases and non-cases of prevalent MetS. We adjusted for age and sex when applicable. Categorical baseline variables were examined using Chi-square tests.

We examined the Shannon index across categories of the SweDGI with the general linear model. The basic model was adjusted for age, sex, study site and total energy intake. In our main model, we also adjusted for smoking, leisure time physical activity and education. We also used models in which we additionally included BMI and/or fibre intake. Shannon index across scores of the individual SweDGI components was examined with the main multivariable model and by adding mutual adjustments for all SweDGI components. The SweDGI components were only weakly to moderately correlated (strongest correlations were seen between fruit & berries and vegetables (rho = +0.36) and between salt and added sugar (rho = –0.36)), indicating that mutual adjustments would not violate the statistical models.

We used logistic regression to examine associations with MetS and performed models including the same covariates as in the general linear models described above and additional models including adjustment for Shannon index when examining associations between the SweDGI and MetS, while including adjustment for the SweDGI when examining the association between the Shannon index and MetS. Our main multivariable model was also used to examine the risks of having components of MetS. All statistical tests were two-sided. Statistical significance was assumed at *P* < 0.05. Associations with *P*-values between 0.05 and 0.10 were reported as tendencies of associations.

Tests for interactions were performed to examine if observed associations with the SweDGI varied by gender, study site, BMI ≤ 25 or > 25 and the Shannon index by adding multiplicative factors of the covariates of interest treated as continuous variables (i.e. Shannon × SweDGI category when examining associations with MetS).

Analyses were performed using both the original SweDGI and the variant that only included energy-adjusted variables as described under the dietary data section above and in Supplementary Tables 1 and 2.

In a sensitivity analysis, we repeated the examination of associations with the Shannon index, using the main multivariable model, in a sample where individuals who used antibiotics during the 6 months preceding data collection were excluded.

### Post hoc analysis

The latest Nordic Nutrition Recommendations (NNR) were launched in June 2023 (34), but the Swedish FBDGs have not yet been updated. Although the coming national guidelines are expected to include additional aspects on the top of NNR2023, such as national culinary habits, health challenges and environmental impacts (34), it is reasonable to assume that the reference values in the updated Swedish FBDGs will be slightly changed to match differences between NNR2023 and NNR2012; slightly lower intakes of meat and sugar (as the latest recommendation includes all free sugar instead of only added sugar), higher vegetable and whole grain intakes, more focus on the intake of fatty fish than on total fish and a stronger emphasis on sufficient intake of low-fat dairy intake to reach recommended calcium intake. We therefore also examined a slightly updated index, as described with a second set of alternatives for some of the index components in Supplementary Table 2, designed to better reflect a diet in line with NNR2023.

## Results

**Fig. 1:**
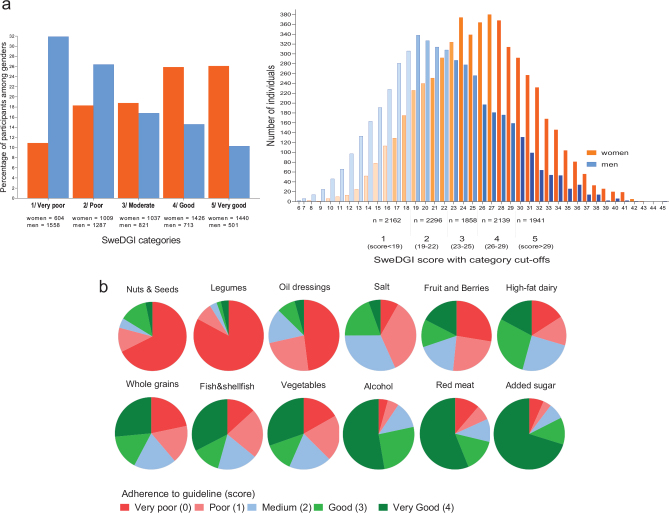
Adherence to the Swedish dietary guidelines as measured with the Swedish dietary guideline index (SweDGI) – distribution of study participants across the SweDGI and its food components. (a) Women showed higher adherence to the overall dietary guidelines. (b) As indicated by green, less than half of the study participants showed good or very good adherence to most of the specific food-based dietary guidelines.

The total SweDGI in the study cohort ranged between scores of 6 and 45 (mean score: 23.8; SD: ±6.2). More women were found in the higher categories of the SweDGI (Fig. 1a), as women had higher scores (range: 8–43, mean: 25.7 ± 5.7) than men (range: 6–45, mean: 21.6 ± 5.9). The percentage of participants estimated to adhere to different food-based guidelines (i.e. score = 4 on the SweDGI components) was lowest for nuts and seeds (3.3%) followed by legumes (3.6%), oil dressings (4.3%) and salt (5.5%), and highest for added sugar (70.1%) followed by meat (56.2%), alcohol (52.6%) and vegetables (30.4%) (Fig. 1b). This pattern was similar in women and men (Supplementary Fig. 2). However, more women than men reached the highest score for all SweDGI components except fish, where a slightly larger part of men reached the highest score (34.3% of the men versus 31.2% of the women). The only SweDGI components on which more than half of the participants got a score above 2 were the ones for added sugar (82.5%), alcohol (78.3%) and meat (71.3%).

### Baseline characteristics

Participants with higher adherence to the Swedish dietary guidelines were found to be older. Fewer among them were smokers, whereas a larger part had a university degree, were born outside Sweden and reported high levels of physical activity. They also had lower BMI, blood pressure, plasma glucose, Haemoglobin A1c (HbA1C), low-density lipoprotein (LDL) cholesterol and triglycerides, and higher HDL cholesterol and gut microbial α-diversity (Shannon index) (Table 1). Similar characteristics to the ones of participants with higher adherence to the guidelines were also seen among those with higher Shannon index and among non-cases of MetS, except that LDL cholesterol did not vary with the Shannon index (Supplementary Table 3), for that fewer with high Shannon index were born outside Sweden, and for that those without MetS were younger and more frequently born in Sweden (Supplementary Table 4a). In addition, participants from Uppsala were more frequently found in the higher quintiles of the Shannon index, and more frequently among those with MetS than among those without MetS. In total, 31% of the study participants were found to have MetS; 34% of the participants from Uppsala and 29% of the participants from Malmö (Supplemental Table 4b). Of the MetS components, high fasting plasma glucose and low HDL cholesterol were more common in Uppsala, whereas high waist circumference was more common in Malmö.

**Table 1:** Baseline characteristics in categories of the SweDGI in 10,396 participants from the SCAPIS Malmö and Uppsala

Characteristics	*n*	Beta ± SE	SweDGI category					*P* for trend^a^ (*P*-value^b^)
			1 6-18 *n* = 2,162	2 19-22 *n* = 2,296	3 23-25 *n* = 1,858	4 26-29 *n* = 2,139	5 30-45 *n* = 1,941	
Age, y	10,396	+0.20 ± 0.03	57.1	57.4	57.6	57.7	57.9	2.2 × 10^-10^
Gender (female), %	10,396		28	44	56	67	75	(7.2 × 10^-45^)
Study centre (Uppsala), %	10,396		43	44	45	47	45	(0.13)
Smokers (current), %	9,974		20	16	12	9	7	(1.7 × 10^-44^)
Leisure-time physical activity (highest level), %	9,806		8	9	10	13	16	(4.3 × 10^-45^)
Education (University degree), %	10,038		31	40	47	54	61	(1.2 × 10^-106^)
Born outside Sweden, %	10,013		12	18	19	22	27	(1.3 × 10^-32^)
BMI, kg/m^2^	10,396	-0.48 ± 0.03	27.9	27.4	27.2	26.5	26.0	4.1 × 10^-50^
Waist, cm	10,395	-1.62 ± 0.08	98.2	96.1	95.1	93.5	91.4	7.9 × 10^-82^
Systolic BP, mm Hg	10,396	-1.27 ± 0.12	127.0	124.9	123.6	123.0	121.6	6.0 × 10^-28^
Diastolic BP, mm Hg	10,396	-0.84 ± 0.07	78.0	76.8	75.9	75.5	74.5	2.5 × 10^-31^
FPG, mmol/L	10,396	-0.03 ± 0.005	5.6	5.5	5.5	5.4	5.4	1.3 × 10^-9^
HbA1c, mmol/mol	10,372	-0.15 ± 0.03	36.1	35.7	35.8	35.5	35.5	1.0 × 10^-6^
Total P-cholesterol, mmol/L	10,394	0.04 ± 0.008	5.66	5.64	5.55	5.60	5.49	2.3 × 10^-7^
P-LDL-C, mmol/L	10,392	-0.03 ± 0.007	3.67	3.66	3.58	3.61	3.52	3.5 × 10^-7^
P-HDL-C, mmol/L	10,396	+0.02 ± 0.003	1.55	1.56	1.57	1.60	1.62	1.7 × 10^-8^
P-TG, mmol/L	10,396	-0.08 ± 0.005	1.45	1.32	1.25	1.20	1.13	2.6 × 10^-44^
Shannon index	9,101	+0.04 ± 0.003	4.01	4.06	4.11	4.13	4.17	2.6×10^-31^

a Adjusted for age and sex when applicable. General linear model.

b *P*-value indicates the difference between any of the categories. Chi-square test.

SweDGI, Swedish dietary guideline index; SCAPIS, Swedish CArdioPulmonary bioImage Study; BMI, body mass index; BP, blood pressure; FPG, fasting plasma glucose; HbA1c, haemoglobin A1c; P-LDL-C, plasma low-density lipoprotein-cholesterol; P-HDL-C, plasma high-density lipoprotein-cholesterol; P-TG, plasma triglycerides.

### Adherence to dietary guidelines and gut microbial α-diversity

**Fig. 2:**
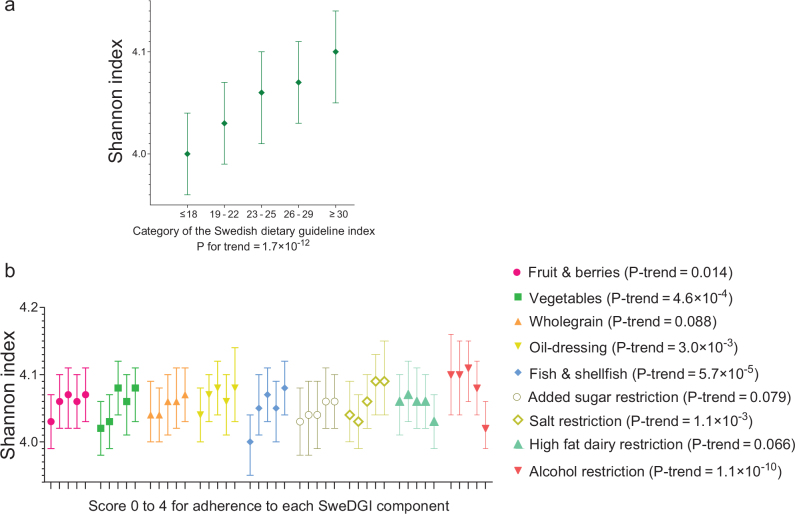
Adherence to the Swedish dietary guidelines and gut microbial α-diversity (Shannon index). (a) Adherence to the overall dietary guidelines, as measured with the Swedish dietary guideline index (SweDGI), was associated with a higher Shannon index (P for trend across SweDGI categories = 1.7 × 10^-12^). (b) Adherence to five of the food-based dietary guidelines was significantly associated with higher α-diversity, that is, those for fruits and berries, vegetables, oil-dressings, fish and salt, and similar tendencies were seen for whole grain (*P* for trend = 0.088) and added sugar (*P* for trend = 0.079). In contrast, adherence to the guideline recommending restricted alcohol intake was associated with lower α-diversity and a similar tendency was seen for the guideline proposing restricted intake of high-fat dairy products.

Higher SweDGI was associated with higher Shannon index (+0.024 per category of the SweDGI; 95% confidence interval [CI]: 0.017, 0.030; *P*-trend across categories = 1.7 × 10^-12^ in the main multivariable model) (Fig. 2a, Supplementary Table 5a). We observed a similar association after adjustment for fibre intake and BMI, as well as when all components included in the dietary index were energy-adjusted (*P* for trend = 5.6 × 10^-12^). The positive association was similar in men and women, and in individuals with BMI ≤ 25 and > 25, but it tended to be slightly stronger in the Uppsala sub-cohort than in the Malmö sub-cohort (*P* for interaction = 0.09) (Supplementary Table 5b).

A higher score on most components of the SweDGI (indicating higher adherence to each FBDG) was also associated with a higher Shannon index before mutual adjustment for the different components (Supplementary Table 6a). All associations went in the same direction both with and without mutual adjustment for other SweDGI components, and five components remained significantly associated with the Shannon index in the mutually adjusted model; higher scores on the components for fruits and berries, vegetables, oil-dressing, fish and salt were then significantly associated with higher Shannon index (Fig. 2b) and tendencies in the same direction were also seen for whole grain (beta = +0.0053, *P* for trend = 0.088) and added sugar (beta = +0.0078, *P* for trend = 0.079), whereas a higher score on the alcohol component was associated with lower Shannon index and such tendency was also seen for the high-fat dairy component. Higher scores on the components for nuts and seeds, legumes and meat were associated with a higher Shannon index before adjustment for other SweDGI components, but not in the mutually adjusted model. Our results were similar in analyses using energy-adjusted intakes for all food components with the score cut-offs expressed as g/MJ, except for that higher score for the nuts (*P*-for trend = 6.4 × 10^-3^) and whole grain (*P*-for trend = 0.025) components were significantly associated with higher Shannon index also in the mutually adjusted models, whereas the association with fruit and berries (*P* for trend = 0.052) was slightly attenuated (Supplementary Table 6b).

### Adherence to dietary guidelines and MetS

**Fig. 3:**
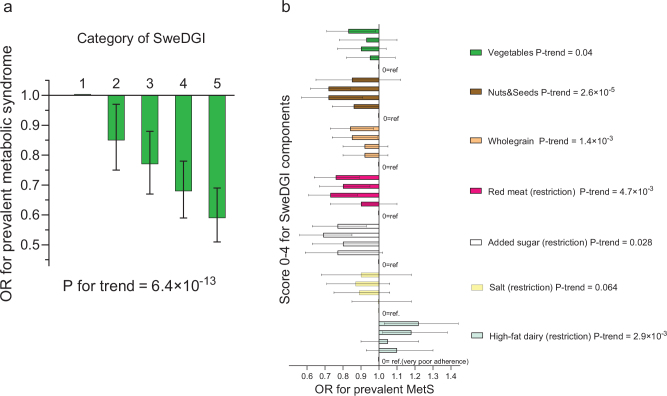
Adherence to the Swedish dietary guidelines and prevalence of metabolic syndrome (MetS). (a) A higher level of adherence to the overall dietary guidelines, as measured with the Swedish dietary guideline index (SweDGI), was associated with a lower prevalence of MetS (*P* for trend = 6.4 × 10^-13^). (b) Adherence to five of the 12 food-based dietary guidelines was inversely associated with the prevalence of MetS, after mutual adjustments for all the food-based dietary guidelines, and a similar tendency was seen for the salt restriction guideline, whereas higher adherence to the guideline proposing restricted intake of high-fat dairy products was associated with a higher prevalence of MetS.

Better overall adherence to the Swedish dietary guidelines, as measured by the SweDGI, was associated with a lower risk of having MetS after adjustment for potential confounders (odds ratio [OR] comparing 5th category SweDGI with the 1st: 0.59; 95% CI: 0.51, 0.69; *P*-trend across categories = 6.4 × 10^-13^) (Fig. 3a and Supplementary Table 7). The association remained significant but was attenuated after additional adjustment for potential mediators, especially after adjustment for BMI (*P* for trend = 1.2 × 10^-5^ after adjustment for BMI, *P* for trend = 3.3 × 10^-11^ after adjustment for the Shannon index, *P* for trend = 2.1 × 10^-9^ after adjustment for fibre intake). We observed similar results using energy-adjusted food components in the SweDGI (*P* for trend = 1.0 × 10^-12^). The association was similar in men and women, but it was stronger in participants with BMI ≤ 25 than with BMI > 25 (*P* for interaction = 0.046) and it tended to be somewhat stronger in the Uppsala sub-cohort than in the Malmö sub-cohort (*P* for interaction = 0.07) (Supplementary Table 8).

The SweDGI was also inversely associated with the risk of having each individual component of MetS (*P*-values for trend ≤0.029) (Supplementary Table 9), in analysis using the main multivariable model. However, higher SweDGI was not associated with the risk of low HDL cholesterol after mutual adjustments for the other MetS components (*P* for trend = 0.83).

A higher score on most components of the SweDGI (indicating better adherence to each FBDG) was associated with a lower risk of MetS in the main multivariable model before mutual adjustments for food components. Better adherence to 10 of the 12 guidelines was associated with lower risk (components for intakes of fruits and berries, vegetables, legumes, nuts and seeds, wholegrain, oil-dressings, meat, added sugar, alcohol and salt) (Supplementary Table 10a). The fish component was not significantly associated with the prevalence of MetS, whereas better adherence to the guideline proposing restriction of high-fat dairy intake was associated with higher prevalence. After mutual adjustment for the SweDGI components, adherence to the six guidelines regarding vegetables, nuts and seeds, wholegrain, meat, added sugar and high-fat dairy remained significant, and we also observed a similar tendency for salt (*P* for trend = 0.064) (Fig. 3b). The associations remained virtually unchanged in analyses using energy-adjusted variables for all food components with score cut-offs expressed as g/MJ (Supplementary Table 10b).

### GM α-diversity and the MetS

**Fig. 4:**
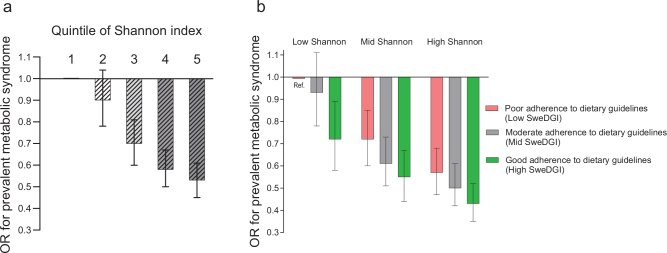
Gut microbial α-diversity (Shannon index) in relation to the prevalence of the metabolic syndrome (MetS), and joint effects of α-diversity and adherence to the dietary guidelines on the prevalence of MetS. (a) Shannon index was inversely associated with the prevalence of MetS (*P* for trend across quintiles = 1.3 × 10^-23^). (b) The lowest prevalence of MetS was observed in the group of individuals found to have both high adherence to the Swedish dietary guidelines index (SweDGI) and high Shannon index (odds ratio [OR]: 0.43; 95% confidence interval [CI]: 0.35, 0.52 compared to the reference group including those with both low SweDGI and low Shannon index). No statistical interaction was seen between the SweDGI and Shannon index on the prevalence of MetS (*P* = 0.82).

A higher Shannon index was associated with a lower risk of having MetS after adjustment for potential confounders (OR comparing the 5th quintile with the 1st:0.53; 95% CI: 0.45, 0.61; *P*-trend across categories = 1.3 × 10^-23^) (Fig. 4a, Supplementary Table 11). Although attenuated, the association remained significant after additional adjustment for BMI (*P* for trend = 5.7 × 10^-8^).

A higher Shannon index was also associated with a lower risk of having each component of MetS (*P*-values for trend across quintiles of Shannon index < 0.001) (Supplementary Table 12), in analysis using the main multivariable model. Although somewhat attenuated, all associations except that with lower risk of hypertension (*P* for trend = 0.08), remained significant after adjustment for other MetS components.

### Interaction between adherence to the dietary guidelines and gut microbial α-diversity on the prevalence of the MetS

The magnitude of the association between tertiles of Shannon index and MetS was similar at low, medium and high SweDGI (Supplementary Table 13), and although the association between SweDGI and MetS appeared to be somewhat stronger among those in the lower Shannon index tertiles, we did not observe any significant interaction (*P* = 0.82), and indications of lower risk of prevalent MetS at higher adherence to the guidelines were seen in all tertiles of the Shannon index (Supplementary Table 14). In the joint effect model, treating low SweDGI and low Shannon index as a reference, we observed the lowest prevalence of MetS in the group of individuals with both high SweDGI and high Shannon index (OR: 0.43; 95% CI: 0.35, 0.52) (Fig. 4b, Supplementary Table 15).

When examining the statistical interaction between the Shannon index and each SweDGI component on MetS, the Shannon index was not found to interact with any of the components (*P*-values for interaction ≥ 0.08) (Supplementary Table 16), except for the high-fat dairy component (*P* for interaction = 0.04). Our results indicated that better adherence to the recommendation proposing limited consumption of high-fat dairy products was associated with a higher prevalence of MetS among those in the higher tertiles of the Shannon index (*P*-values for trend = 0.02 and 0.01, respectively), but not among those in the lowest tertile of Shannon index (*P* for trend = 0.65) (Supplementary Table 17). A higher Shannon index was observed to be associated with a lower prevalence of MetS at all three levels of adherence to the high-fat dairy guideline, although somewhat less strongly among those with good adherence (Supplementary Table 18).

### Sensitivity analysis

We observed similar positive associations between the SweDGI and Shannon index when excluding individuals who used antibiotics during the last 6 months before baseline examination (*P* for trend across the categories of the SweDGI = 5.1 × 10^-13^) as in the original study sample and the inverse association between Shannon index and prevalence of MetS remained similar (*P* = 5.3 × 10^-20^). In addition, the previously observed significant associations between components of SweDGI and the Shannon index remained virtually unchanged in the sensitivity analysis, whereas two of the components that only showed tendencies of associations in the mutually adjusted model in the original sample were significantly associated with Shannon index in the sensitivity analysis: whole grain (beta = +0.01; *P* for trend = 0.046) and restricted high-fat dairy intake (beta = –0.01; *P* for trend = 0.012). Finally, the tendency of association between added sugar and the Shannon index in the original study sample was not seen in the sensitivity analysis (*P* for trend = 0.56).

### Post hoc analysis

For post hoc analysis, we created a slightly modified SweDGI, as described in Supplementary Table 2, that also reflects expected revisions in the upcoming version of the Swedish dietary guidelines. We observed similar associations between adherence to the modified index and both higher Shannon index (+0.021 per category of the SweDGI; 95% CI: 0.014, 0.027; *P*-trend across categories = 3.9 × 10^-9^) and lower prevalence of MetS (OR comparing 5th category SweDGI with the 1st: 0.61; 95% CI: 0.52, 0.72; *P*-trend across categories = 4.1 × 10^-12^) as with our original SweDGI.

## Discussion

In this large study, rather poor adherence to the Swedish dietary guidelines was seen among 50–64 years old people in two different Swedish city areas. Our results indicated that better overall adherence to the dietary guidelines, as measured by SweDGI, was associated with a more diverse GM, and that most of the 12 specific FBDGs contributed to this finding. Adherence to five of the guidelines was *per se* significantly associated with higher α-diversity, that is, those for fruits and berries, vegetables, oil-dressings, fish and salt, and similar tendencies were seen for those for whole grain and added sugar. Higher overall adherence to the guidelines was also associated with a lower prevalence of MetS. Adherence to guidelines regarding several different food groups was found to contribute to this finding. Higher adherence to the guideline promoting vegetable consumption was significantly associated with both a more diverse GM and lower prevalence of MetS, and similar indications were seen for whole grain, added sugar and salt. Higher gut microbial α-diversity was in turn also associated with lower prevalence of MetS. However, adjustment for the Shannon index did not substantially change the association between adherence to the guidelines and MetS, suggesting that it is not to a major part mediated by the gut microbial α-diversity. Finally, the prevalence of MetS was less than half as high in the group of study participants with both the best adherence to the dietary guidelines and the most diverse GM compared with prevalence in the group with the poorest adherence and the least diverse GM. The association between gut microbial α-diversity and MetS was consistent for those with low and high adherence to the guidelines and there was no statistical interaction between the SweDGI and Shannon index.

Our results remained virtually unchanged after a slight modification of SweDGI intended to also capture estimated revisions of the Swedish FBDGs expected to be launched in 2024, indicating that our index also will be applicable to the upcoming version of the guidelines.

Low adherence to national dietary guidelines has also been observed in other Swedish or high-income populations (35, 36). Previous studies examining healthy dietary patterns and their components in relation to gut microbial α-diversity, which in contrast to our study have utilized 16S-based GM analyses, have in general also indicated positive associations (29, 37–41). Both the Healthy Eating Index, which reflects adherence to the dietary guidelines for Americans, and the Mediterranean Dietary Score, were for example positively associated with the Shannon index in the TwinsUK cohort (29). Similarly, short-term adherence to a healthy plant-based dietary pattern was associated with higher gut microbial α-diversity in a Chinese study, although they did not observe a distinct stepwise higher Shannon index per healthier diet category as in our study (37). A higher Shannon index was also seen across three categories of a diet diversity score, although only the difference in Shannon index between the two lower categories could be replicated in the validation cohort of that study (38). Furthermore, Vangay et al. found that the Shannon index was lower among two minority groups from Thailand residing in the United States (US) compared to that among the same ethnic groups residing in Thailand and that the lowest Shannon index was found among the second-generation US residents (41). They also identified differences in dietary patterns between the second-generation US residents and the group in Thailand. By using deep metagenomic sequencing data, they found that the microbiome of immigrants was characterized by loss of functions related to the degradation of complex carbohydrates, whereas functions related to sucrose degradation were enriched. That observation could reflect dietary changes where fibre-rich plant foods are replaced by sugar-rich foods and may correspond to dietary differences among those with high vs. low SweDGI in our study. In contrast to findings in most studies, healthy dietary patterns were not associated with the Shannon index in the PREDICT 1 study with microbiota data from deep metagenomic sequencing like ours (42), which for example could be explained by differences in the level of adherence and distribution of study participants across the dietary indices. Interestingly and in line with our findings, Maskarinec et al. found that the Healthy Eating Index components for fruits, vegetables, whole grains and salt were associated with higher α-diversity (40). In addition, somewhat surprisingly they reported that a fatty acid ratio in line with their guidelines was associated with lower α-diversity, which could correspond to our finding that restricted consumption of high-fat dairy products was linked to lower α-diversity. Maskarinec et al. did not have fish intake as a separate index component, but our positive association between fish intake and α-diversity may reflect a higher intake of omega-3 fatty acids, which has been associated with a higher α-diversity in animal studies. However, evidence from studies in humans linking omega-3 fatty acids to α-diversity is missing (43). Our results also confirm that both dietary patterns and gut microbial α-diversity are connected to metabolic health (44, 45). The MetS prevalence of 31% in our study is comparable with the prevalence of 28–29% observed in adults from high-income countries using a similar MetS definition (3), considering that our study only included adults aged 50–65 years. The Shannon index was found to be inversely associated with MetS independently of overall adherence to the dietary guidelines, and although it has been suggested that the GM may modify associations between diet and metabolic health (44, 46–49), higher overall adherence to the dietary guidelines was linked to lower prevalence of MetS independently of Shannon index. Our results also showed that adherence to several of the specific food-based guidelines included in the index *per se* was associated with a lower prevalence of MetS independently of α-diversity. Previous studies have also shown a lower prevalence of MetS at high intakes of whole grains (50), and nuts and seeds (51), although it was argued that more evidence from prospective studies is needed. The potential benefits of vegetable consumption on MetS, as indicated in our study, seem to be less conclusive in previous studies (52, 53). In contrast, some previous studies have observed inverse associations between fruit consumption and MetS (52, 53), whereas in our study this association did not remain after adjustment for the other SweDGI components. Meat consumption has in line with our findings been associated with a higher prevalence of MetS (54), and similar to our findings associations have been observed for intakes of added sugar or sugar-rich food (55, 56), as well as for salt intake that has been associated with future development of MetS (57). In our study, the Shannon index was only found to modify associations between high-fat dairy consumption and MetS; better adherence to the guideline proposing lower consumption of high-fat dairy products was actually related to a higher prevalence of MetS in participants with moderate to high levels of α-diversity, but not among those with low α-diversity. To the best of our knowledge, such interplay has not been examined in previous studies. Despite evidence supporting the recommendations of limited consumption of foods high in saturated fat such as high-fat dairy products (58), and although most prospective studies also indicate favourable effects of low-fat dairy on MetS (59), a few previous observational studies have indeed indicated lower prevalence of type 2 diabetes among individuals with high consumption of high-fat dairy products or dairy fats (7, 60). Our observations should however be interpreted with caution as we cannot exclude that it is a result of reverse causation. Replacement of high-fat dairy products with low-fat alternatives could be an easy step towards more healthy food choices for persons changing dietary habits due to health-related reasons. Reported intake of dairy products may therefore be less representative of long-term consumption that may have contributed to the development of MetS, than the reported intake of other foods.

There are several plausible biological mechanisms that may explain our findings. As dietary sources of fermentable fibres, fruits, vegetables, whole grains, and nuts and seeds can provide substrates for several short-chain fatty acid (SCFA)-producing gut bacteria and affect their abundancy (61) and thereby also the overall gut microbial composition and diversity. In addition, plant foods provide flavonoids that also can be metabolized by certain gut bacteria. Some of the microbiota-produced flavonoid metabolites have anti-inflammatory effects and may thereby balance circulating lipid levels, whereas others may have antihypertensive properties. Flavonoids may by this means promote cardiometabolic health (62, 63). Salt intake may influence the composition of the GM and induce dysbiosis, which may partly be due to that some bacteria carry a salt tolerance gene whereas others do not (64). Restricted salt intake may favour SCFA-producing species and increase circulating SCFAs, which almost entirely originate from our gut bacteria (65, 66). SCFAs may prevent weight gain via stimulation of diet-induced satiety hormones, such as glucagon-like peptide 1 and leptin. They are also involved in glucose and lipid metabolism, as well as in blood pressure regulation and may consequently improve cardiometabolic health (65). Intake of fish and oil dressings contain omega-3 fatty acids that like other polyunsaturated fatty acids can be metabolized by the GM (67) and it has been suggested that foods rich in omega-3 fatty acids may improve the gut bacterial composition (68), for example suppressing Gram-negative bacteria with surface lipopolysaccharides that could trigger inflammation (69).

A strength of our study is the large population-based design. The size is especially notable in comparison to that of most earlier studies with GM data, and our high-quality data from shotgun metagenomics is also an advantage. Moreover, we are not aware of any study that so extensively examined adherence to all food components included in the Swedish dietary guidelines (35, 70). The dietary data collected, using a validated dietary assessment method, is also valuable (23, 26–28), as well as data from two different regions in Sweden were included. In addition, we had extensive information on potential confounders. Nevertheless, we cannot exclude the occurrence of residual confounding and that unmeasured environmental factors could have influenced our findings. Furthermore, measurement error in self-reported diet is a problem in nutritional epidemiology and may be of special concern in overweight individuals. Indeed, we observed the strongest inverse associations between SweDGI and MetS in individuals with BMI ≤25, but as associations with Shannon index did not differ with BMI status, the weaker associations between SweDGI and MetS in overweight individuals may rather be explained by other characteristics of overweight individuals that could have outweighed the importance of diet for MetS. Moreover, due to dietary measurement errors, we cannot relate the observed associations to exact intake levels, although most of the SweDGI components were based on absolute intakes in accordance with how the dietary guidelines are expressed. However, we observed similar associations using energy-adjusted variables, and in the sensitivity analysis excluding users of antibiotics, as well as with the slightly modified version of the SweDGI to capture expected updates of upcoming dietary guidelines, which illustrates the robustness of our results. We are aware that absolute salt intake is difficult to estimate in observational studies and our decision to include salt intake as a component in our index was thoroughly considered. To minimize the risk that the strong correlation between salt intake and intake of most foods, and consequently energy intake, would confound our results, we decided to include energy-standardized salt intake. In addition, we could incorporate information on salting at the table to the salt component of SweDGI since MiniMeal-Q, in contrast to most food frequency questionnaires (FFQ), includes a question about salting at the table. Although we observed significant associations in the expected direction with gut microbial diversity and/or MetS for nine of the 12 dietary guidelines, the fact that only a few of the study participants got the highest scores on the SweDGI components is a limitation. Consequently, null findings related to legume intake may simply reflect the low number of participants reaching potential threshold levels and a lack of power to evaluate associations. Intake of oil dressings was chosen as an index component, although it only partly captures the intake of healthy food fats. However, we observed that a higher score of the oil-dressing component was associated with a more diverse GM, which supports the relevance of including it as a component of the SweDGI. The cross-sectional design of our study is a major limitation, but data on incident disease is unfortunately not yet available in SCAPIS. Still, by showing that better adherence to dietary guidelines is associated with both lower prevalence of MetS and a more diverse GM, in line with results from previous studies (40, 44), we identify SweDGI and its components as valuable tools for examination of dietary quality-disease associations in future prospective studies. The index could also be used to adjust for diet quality when examining association with other exposures than diet.

## Conclusion

In two sub-cohorts within the large multicentre SCAPIS cohort, we created a new dietary index to quantify overall adherence to the Swedish dietary guidelines. We demonstrated strong associations between higher adherence to the guidelines and a richer and more even GM and better metabolic health. The inverse association between gut microbial richness and MetS was consistent at low and high adherence to the guidelines. Future longitudinal studies examining the interplay between diet and specific gut microbial species, instead of overall richness, are warranted. Together with our findings, they could contribute deeper knowledge to consider when investigating connections between diet and metabolic health and when designing future dietary guidelines with the potential to improve personalized dietary counselling in clinical practice.

## Supplementary Material


