# Supplementary material for: A Swedish dietary guideline index, gut microbial α-diversity and prevalence of metabolic syndrome – observations in the Swedish CArdioPulmonary bioImage Study (SCAPIS)
Source: Food Nutr Res. 2024 Nov 28;68:10.29219/fnr.v68.10547. doi: 10.29219/fnr.v68.10547 (PMC11650442; doi:10.29219/fnr.v68.10547)
Supplement: Supplementary file 1 [file FNR-68-10547-s1.docx]

**Supplementary data**

**Supplementary figures**

*Supplementary Figure 1.* Flowchart of study samples from the SCAPIS Malmö and Uppsala study sites.

*Supplementary Figure 2.* Proportion of women and men estimated to adhere to the different dietary guidelines.

**Supplementary tables**

*Supplementary Table 1.* Index components based on food-based dietary guidelines. Description of overall structure for each component including five levels of adherence.

| **Level of adherence** | **Points received** |
| --- | --- |
| *Very good adherence*  > or < defined reference level^a^ | 4 |
| *Good adherence*  <±25% from reference level | 3 |
| *Moderate adherence*  ±25-50% from reference level | 2 |
| *Poor adherence*  ±50-75% from reference level | 1 |
| *Very poor adherence*  >±75% from reference level | 0 |

a Encourage intakes: Cut-offs at -25%, -50% and -75%, from reference level,

restricted intakes: Cut-offs at +25%, +50% and +75%, from reference level.

*Supplementary Table 2.* Description of the 12 components of the Swedish Dietary Guideline Index (SwDGI) is shown in green (encouraged intakes) and red (restricted intakes). Alternative components based on energy adjusted intakes are shown with paler shading. A second set of alternatives for some of the components for post hoc-analysis of an updated index to better match NNR2023 are without shading.

| **Component** | **Component description** | **Category ranges of component** | | | | |
| --- | --- | --- | --- | --- | --- | --- |
| *Encouraged:* |  | 0 | 1 | 2 | 3 | 4 |
| **1. Fruits and berries** (g/d) | Based on that the guideline proposing 500 g fruits and vegetables states that the intake should include different kinds of fruits and vegetables <https://www.fao.org/3/az907e/az907e.pdf>., we have separate components for fruits and vegetables with a target level of **250g** for each component. Separate components for fruits and vegetables will also reflect that a high intake of plant-based foods is emphasized in the dietary guidelines due to health and climate aspects. Plant-based components could therefore have large weight in the total score. | ≤62.5 | >62.5-125 | >125-187.5 | >187.5-250 | >250 |
| Fruit and berries  (g /MJ) * | 250g/10 MJ corresponds to 25g/MJ | ≤6.25 | >6.25-12.5 | >12.5-18.75 | >18.75-25.0 | >25.0 |
| **2. Vegetables**  (g/d) | Vegetables (250g) were divided into “vegetables non-legumes” (target=**200g**) and “legumes” (target=50g) as it is stressed in the dietary guideline report that legume consumption is encouraged due to its lower climate impact compared to animal protein sources and due to that it is stated that legumes together with onion, cruciferous vegetables and root vegetables are especially important for healthy food habits. We therefore estimated that legumes could contribute to 20% of the total vegetable intake, and that onion, cruciferous vegetables, roots (except potatoes) and other vegetables could contribute to 20% each of the total vegetable intake, i.e., vegetables non-legumes contribute to 80% of total vegetable intake. | ≤50 | >50-100 | >100-150 | >150-200 | >200 |
| Vegetables  (g/MJ) * | 200 g/10 MJ corresponds to 20 g/MJ | ≤5.0 | >5.0-10.0 | >10.0-15.0 | >15.0-20.0 | >20.0 |
| *Post hoc*  *Vegetables (g/d)* | Vegetables non-legumes was increased to 250 g, as pulses should not be included in advice regarding fruits and vegetables according to NNR2023. | *≤62.5* | *>62.5-125* | *>125-187.5* | *>187.5-250* | *>250* |
| **3. Legumes**  (g/d) | Legumes was decided to be a specific component (target=**50g**) as it is stressed in the Dietary guideline report that legume consumption is encouraged due to its lower climate impact compared to animal protein sources and due to that it is stated that legumes together with some other vegetables are especially important for healthy food habits. We estimated that legumes could contribute to 20% of the total vegetable intake, and that other vegetables could contribute to 80% of the total vegetable intake. | ≤12.5 | >12.5-25 | >25-37.5 | >37.5-50 | >50 |
| Legumes  (g/MJ) * | 50 g/10MJ corresponds to 5g/MJ | ≤1.25 | >1.25-2.5 | >2.5-3.75 | >3.75-5.0 | >5.0 |
| **4. Nuts**  (g/d) | Based on the guideline of 2 tbsp nuts and seeds per day, the target level was set at **20g** per day (assuming 65g nuts and seeds/dl) | ≤5g | >5-10 | >10-15 | >15-20 | >20 |
| Nuts  (g/MJ) * | 20 g/10MJ corresponds to 2g/MJ | ≤0.5g | >0.5-1.0 | >1.0-1.5 | >1.5-2.0 | >2.0 |
| **5. Whole grain**  (g/MJ) | In the dietary guidelines it is stated that whole grain products of pasta, bread, grains and rice should be consumed instead of processed products. There is no advice regarding absolute intake level, but it is mentioned that an intake corresponding to 75 grams of whole grains per 10 MJ is considered to be an appropriate amount. The target was therefore set at **7.5** g per MJ. | ≤1.875 | >1.875-3.75 | >3.75-5.625 | >5.625-7.5 | >7.5 |
| *Post hoc*  *Whole grain (g/d)* | Based on the advice to have an intake of 90 g per day in NNR2023 | ≤22.5 | >22.5-45 | >45-67.5 | >67.5-90 | >90 |
| **6. Oil-dressing**  (g/d) | The component reflecting adherence to the guideline regarding healthy food-fats in the SweDGI was restricted to the part in the guideline where it is stated that use of oil-dressings should be encouraged. The reason is that the FFQ did not give information on frequencies of fats used for cooking (only yes/no) and that it was possible to select several options for cooking fats and fat-spreads (yes/no), and it was therefore not possible to get information on the most commonly used fats.  The target was set to one tablespoon per day (**13.5 g**). | ≤3.4 | >3.4-6.8 | >6.8-10.1 | >10.1-13.5 | >13.5 |
| Oil-dressing  (g/MJ) * | 13.5 g /10 MJ corresponds to 1.35g/MJ | ≤0.34 | >0.34-0.68 | >0.68-1.01 | >1.01-1.35 | >1.35 |
| *Post hoc:*  *Alpha-linolenic acid (ALA) (g/MJ)* | Based on advice to consume a sufficient amount vegetable oil to reach an intake of 1.3g ALA per 10 MJ, which corresponds to 0.13g/MJ. | ≤0.0325 | >0.0325-0.065 | >0.065-0.0975 | >0.0975-0.13 | >0.13 |
| **7. Fish**  (g/d) | Based on the dietary guideline indicating fish 2-3 times per week and a portion size of 115g, the target intake was set to **40g/d**. | ≤10 | >10-20 | >20-30 | >30-40 | >40 |
| Fish  (g/MJ) * | 40 g /10 MJ corresponds to 4g/MJ | ≤1.0 | >1.0-2.0 | >2.0-3.0 | >3.0-4.0 | >4.0 |
| *Post hoc:*  *Fish, fatty*  (g/d) | Based on advice in NNR 2023 of 300(-450g)/week, where of at least 200g fatty fish, half of the points for fish comes from intake of fatty fish and half from total fish | ≤7.25 | >7.25-14.5 | >14.5-21.75 | >21.75-29 | >29 |
| *Fish, total*  (g/d) |  | ≤10.75 | >10.75-21.5 | >21.5-32.25 | >32.25-43 | >43 |
| *Post hoc:*  *Low-fat dairy (portions/d)* | Based on recommendation in NNR 2023 of 350-500 (=1.75-2.5 portions) low-fat milk and dairy.  Low-fat dairy brings half of the dairy points (i.e., 0-2 p) and high-fat dairy half. (0-2 p instead of 0-4 p). See below for high-fat dairy. | *≤0.438* | *>0.438-0.875* | *>0.875-1.312* | *>1.312-1.75* | *>1.75* |
|  |  |  |  |  |  |  |
| *Restricted:* |  |  |  |  |  |  |
| **8. Red meat**  (g/d) | Based on the dietary guideline indicating that the intake of red and processed meat should be restricted to no more than 500g/week, the target was set to **71g per day** or less. | >125 | >107-125 | >89-107 | >71-89 | ≤71 |
| Red meat  (g/MJ) * | 71g/d for a person with an energy intake of 10MJ corresponds to 7.1g/MJ | >12.5 | >10.7-12.5 | >8.9-10.7 | >7.1-8.9 | ≤7.1 |
| Post hoc:  Red meat | Slightly changed reference value of 50g/d based on the recommendation of 350g/week in NNR 2023. | >87.5 | >75-87.5 | >62.5-75 | >50-62.5 | ≤50 |
| **9. High-fat dairy** (portions/d) | Based on the dietary guideline recommending replacement of high fat dairy products, this component reflects intake portions of high fat dairy products. Since there is no recommendation regarding exact intake levels, we set the target level to **≤1portion** per day. | >4 | >3-4 | >2-3 | >1-2 | ≤1 |
| High-fat dairy (portions/MJ) * | ≤1portion per 10MJ corresponds to ≤0.1portion/MJ | >0.4 | >0.3-0.4 | >0.2-0.3 | >0.1-0.2 | ≤0.1 |
| *Post hoc:*  *High-fat dairy*  *(portions/d)* | Low-fat dairy brings half of the dairy points (i.e., 0-2p) and high-fat dairy half. (0-2p instead of 0-4p). See above for low-fat dairy. | >4 | >3-4 | >2-3 | >1-2 | ≤1 |
|  |  |  |  |  |  |  |
| **10. Added sugar**  (E%) | There is a guideline indicating limited consumption of sugar in general and particularly from sweet drinks. However, since there are no exact recommendations regarding intake levels of SSB or other sugar-rich foods, added sugar was chosen as a marker of sugar-rich foods.  According to NNR 2012, the intake of added sugar should only contribute to at most 10 percent of the energy intake. The reference level was therefore set to **≤ 10 non-alcohol E%**, although some authorities including WHO and Public health England have proposed even lower reference levels. | >17.5 | >15-17.5 | >12.5-15 | >10-12.5 | ≤10 |
| *Post hoc:*  *Free sugar*  **(E%)** | Slightly changed to apply the sugar recommendation of 10 E% to intake of free sugar instead of added sugar, i.e., to also count sugar from juice and thereby make the recommendation somewhat stricter. | >17.5 | >15-17.5 | >12.5-15 | >10-12.5 | ≤10 |
| **11. Alcohol**  (E%) | In NNR 2012 below 5 E% was recommended, but since it was stated in the report on dietary guidelines 2015 that all intakes could increase the risk of cancer, we have set the target level based on 50% of the recommended level in NNR 2012, i.e., **<2.5 E%**. For a woman with an energy intake of 2000 kcal, 2.5 E% corresponds to a daily intake of 7.1 g alcohol/d (~0.5 glasses of wine per day or a bottle of light beer). | >10 | >7.5-10 | >5-7.5 | >2.5-5 | ≤2.5 |
| **12. Salt**  (g/MJ) | The guideline regarding salt intake, includes advice both regarding intake of foods with low salt content, as well as to put less salt on the food. Adherence to the salt guideline was therefore based on two criteria.  a/ The estimated intake from all foods consumed except salt added at table:  According to NNR 2012 intake from food excluding salting contributes to up to 90% of our total salt intake. Adherence to this first criterium therefore gave 0-3 pts, instead of 0-4 pts.  According to the Nordic nutrition recommendations the salt intake should not exceed 6 g per day. Salt intake is difficult to measure using self-reported intake and as all food contribute salt, the salt intake was more strongly correlated to energy intake (rho=0.83), compared to the other dietary intakes with recommended intakes expressed as absolute intakes (rho=0.07-0.30). As measurement errors of dietary intakes can be reduced by using energy adjusted dietary intakes (Willett et al, Nutritional epidemiology 2013), we decided to express the reference level for salt intake as g/MJ. The reference level for salt from foods was consequently set to **0.6 g/MJ** (corresponding to 6 g per day at a daily energy intake of 10 MJ). The other cut-offs were set to 0.75, 0.9 g/MJ.  b/ Salt usually not added at table (based this answer option to a specific question in MiniMeal-Q regarding salting at table).  Adherence to this criterium gave 0-1 pts (missing answer on this question (n=55) were interpreted as no salting at table and gave 1 p). | >0.9 | >0.75-0.9 | >0.6-0.75 | ≤0.6 | Extra point=No salting at table |

*Component for score based on energy-adjusted components. Based on the assumption that the reference values correspond to an energy intake of 10MJ.

*Supplementary Table 3.* Baseline characteristics across quintiles of Shannon index in 10,396 men and women from the SCAPIS Malmö and Uppsala centra

|  |  |  | Shannon quintile | | | | |  |
| --- | --- | --- | --- | --- | --- | --- | --- | --- |
|  | n | Beta±SE | 1 | 2 | 3 | 4 | 5 | P for trend^a^  (P-value^b^) |
| Age, y | 9,101 | +0.13±0.03 | 57.3 | 57.4 | 57.4 | 57.7 | 57.8 | 4.2×10^-5^ |
| Gender (female), % | 9,101 |  | 48 | 49 | 54 | 56 | 58 | (3.5×10^-11^) |
| Study centre (Uppsala), % | 9,101 |  | 44 | 46 | 48 | 52 | 58 | (1.2×10^-18^) |
| Smokers (current), % | 8,723 |  | 18 | 14 | 13 | 11 | 8 | (2.4×10^-19^) |
| Leisure-time physical activity (highest level), % | 8,595 |  | 8 | 11 | 11 | 12 | 15 | (1.2×10^-36^) |
| Education (University degree), % | 8,787 |  | 36 | 42 | 48 | 51 | 58 | (7.4×10^-39^) |
| Born outside Sweden, % | 8,770 |  | 24 | 20 | 19 | 16 | 15 | (4.7×10^-12^) |
| BMI, kg/m^2^ | 9,101 | -0.56±0.03 | 28.2 | 27.6 | 26.9 | 26.6 | 25.9 | 8.5×10^-68^ |
| Systolic BP, mm Hg | 9,101 | -0.57±0.12 | 125.2 | 124.0 | 123.2 | 122.8 | 122.9 | 9.4×10^--7^ |
| Diastolic BP, mm Hg | 9,101 | -0.36±0.07 | 76.7 | 76.0 | 75.6 | 75.3 | 75.3 | 6.1×10^-7^ |
| FPG, mmol/L | 9,101 | -0.03±0.006 | 5.57 | 5.54 | 5.50 | 5.47 | 5.46 | 4.0×10^-7^ |
| HbA1c, mmol/mol | 9,082 | -0.30±0.03 | 36.5 | 36.0 | 35.7 | 35.5 | 35.2 | 1.2×10^-21^ |
| Total P-cholesterol, mmol/L | 9,099 | +0.03±0.008 | 5.55 | 5.57 | 5.59 | 5.64 | 5.69 | 5.0×10^-6^ |
| P-LDL-C, mmol/L | 9,097 | +0.01±0.007 | 3.60 | 3.62 | 3.62 | 3.64 | 3.63 | 0.30 |
| P-HDL-C, mmol/L | 9,101 | +0.04±0.003 | 1.50 | 1.52 | 1.58 | 1.60 | 1.65 | 2.7×10^-33^ |
| P-TG, mmol/L | 9,101 | -0.09±0.002 | 1.48 | 1.36 | 1.24 | 1.19 | 1.13 | 1.1×10^-57^ |

a Adjusted for age and sex when applicable. General linear model.

b P-value indicates difference between any of the categories. Chi-square test

BP, blood pressure; FPG, fasting plasma glucose; HbA1c, Haemoglobin A1c; P-LDL-C, Plasma low-density lipoprotein-cholesterol; P-HDL-C, Plasma high-density lipoprotein-cholesterol; P-TG, Plasma triglycerides.

*Supplementary Table 4a.* Baseline characteristics according to prevalence of the Metabolic syndrome in

10,396 men and women from the SCAPIS Malmö and Uppsala centra

|  | n |  | Non cases | Cases | P-value^a^ |
| --- | --- | --- | --- | --- | --- |
| Age, y | 10,396 |  | 57.1 | 58.4 | 2.8×10^-43^ |
| Gender (female), % | 10,396 |  | 56 | 46 | 1.1×10^-20^ |
| Study centre (Uppsala), % | 10,396 |  | 43 | 49 | 1.6×10^-8^ |
| Smokers (current), % | 9,974 |  | 12 | 16 | 1.8×10^-17^ |
| Leisure-time physical activity (highest level), % | 9,806 |  | 15 | 6 | 2.2×10^-73^ |
| Education (University degree), % | 10,038 |  | 50 | 40 | 1.5×10^-21^ |
| Born outside Sweden, % | 10,013 |  | 19 | 21 | 0.004 |
| BMI, kg/m^2^ | 10,396 |  | 25.8 | 29.7 | <0.001 |
| Systolic BP, mm Hg | 10,396 |  | 120.1 | 132.8 | <0.001 |
| Diastolic BP, mm Hg | 10,396 |  | 73.8 | 81.4 | 9.8×10^-303^ |
| FPG, mmol/L | 10,396 |  | 5.32 | 5.87 | 5.8×10^-276^ |
| HbA1c, mmol/mol | 10,372 |  | 35.1 | 37.2 | 2.1×10^-131^ |
| Total P-cholesterol, mmol/L | 10,394 |  | 5.60 | 5.57 | 0.12 |
| P-LDL-C, mmol/L | 10,392 |  | 3.60 | 3.64 | 0.023 |
| P-HDL-C, mmol/L | 10,396 |  | 1.69 | 1.35 | <0.001 |
| P-TG, mmol/L | 10,396 |  | 1.07 | 1.73 | <0.001 |

a General linear model for continuous variables, adjusted for age and sex when applicable. Chi-square test for categorical variables.

BP, blood pressure; FPG, fasting plasma glucose; HbA1c, Haemoglobin A1c; P-LDL-C, Plasma low-density lipoprotein-cholesterol;

P-HDL-C, Plasma high-density lipoprotein-cholesterol; P-TG, Plasma triglycerides.

*Supplementary Table 4b.* Prevalence of the Metabolic syndrome and its components depending on study site in

10,396 SCAPIS participants from Malmö and Uppsala

|  |  | All | Malmö | Uppsala | P-value^c^ |
| --- | --- | --- | --- | --- | --- |
|  | n | Cases | Cases | Cases |  |
| Metabolic syndrome | 10,396 | 31.0 | 28.7 | 33.8 | <0.001 |
| Waist, high  (men: ≥ 94cm, women: ≥ 80) | 10,396 | 75.5 | 76.8 | 73.9 | <0.001 |
| Blood pressure, high^a^  (SBP ≥ 130/DBP ≥ 85 mmHg) | 10,396 | 42.0 | 41.9 | 42.2 | 0.70 |
| Glucose (≥ 5.6 mmol/L) | 10,396 | 42.1 | 35.7 | 50.0 | <0.001 |
| Triglycerides, high^b^  (≥ 1.7 mmol/L) | 10,396 | 24.0 | 24.5 | 23.5 | 0.22 |
| HDL-cholesterol, low  (women: ≤ 1.29 mmol/L, men: 1.03) | 10,396 | 10.3 | 8.6 | 12.3 | <0.001 |

a, Or treatment with antihypertensive drugs.

b, Or treatment with lipid-lowering drugs.

c, Chi-square test.

*Supplementary Table 5a.* Shannon index in categories of the Swedish dietary guideline index (SweDGI) in 9,101 SCAPIS participants from SCAPIS Malmö and Uppsala

|  |  | Category of SweDGI | | | | | |  |
| --- | --- | --- | --- | --- | --- | --- | --- | --- |
| All | Beta^a^ | 1  ≤ 18 | 2  19-22 | 3  23-25 | 4  26-29 | 5  ≥ 30 | P-trend^b^ | P-trend^b^ energy-adjusted SweDGI |
| Basic^c^ | +0.040 | 4.00  3.99, 4.03 | 4.06  4.04, 4.08 | 4.10  4.08, 4.12 | 4.13  4.11, 4.15 | 4.17  4.15, 4.19 | 1.2×10^-33^ | 1.1×10^-32^ |
| Main multivariable model^d^ | +0.024 | 4.00  3.96, 4.04 | 4.03  3.99, 4.07 | 4.06  4.02, 4.10 | 4.07  4.03, 4.11 | 4.10  4.06, 4.14 | 1.7×10^-12^ | 5.6×10^-12^ |
|  |  |  |  |  |  |  |  |  |
| Multivariable model with BMI | +0.021 | 4.01  3.97, 4.05 | 4.04  4.00, 4.08 | 4.07  4.02, 4.11 | 4.07  4.03, 4.11 | 4.10  4.05, 4.14 | 9.0×10^-10^ | 4.0×10^-9^ |
|  |  |  |  |  |  |  |  |  |
| Multivariable model with fibre | +0.028 | 3.99  3.95, 4.03 | 4.03  3.99, 4.07 | 4.06  4.02, 4.10 | 4.07  4.03, 4.12 | 4.11  4.06, 4.15 | 1.0×10^-10^ | 5.6×10^-10^ |
|  |  |  |  |  |  |  |  |  |
| Multivariable model with BMI + fibre | +0.026 | 4.00  3.96, 4.04 | 4.04  3.99, 4.08 | 4.07  4.03, 4.11 | 4.08  4.04, 4.12 | 4.11  4.07, 4.15 | 3.6×10^-9^ | 2.9×10^-8^ |

^a^Beta indicates mean difference per category of SweDGI.

^b^General linear model.

^c^Adjusted for age, sex, study site, and total energy intake.

^d^Adjusted for age, sex, study site, total energy intake, smoking and leisure time physical activity and education.

*Supplementary Table 5b.* Shannon index in category of Swedish dietary guideline index (SweDGI) in site, gender and BMI stratified analysis, in 4,567 Malmö-participants and 4,534 Uppsala participants, 4,830 women and 4,271 men, and 3252 participants with BMI ≤25 and 5,849 participants with BMI >25 from SCAPIS

|  |  | Category of SweDGI | | | | | |  |
| --- | --- | --- | --- | --- | --- | --- | --- | --- |
| All | Beta | 1  ≤ 18 | 2  19-22 | 3  23-25 | 4  26-29 | 5  ≥ 30 | P-trend^a^ |  |
| Malmö |  |  |  |  |  |  |  |  |
| Main multivariable model | +0.019 | 4.00  3.91, 4.03 | 4.01  3.95, 4.07 | 4.01  3.95, 4.07 | 4.02  3.97, 4.08 | 4.06  4.00, 4.12 | 5.2×10^5^ |  |
| Uppsala |  |  |  |  |  |  |  |  |
| Main multivariable model | +0.028 | 4.04  3.97, 4.10 | 4.06  4.00, 4.12 | 4.11  4.05, 4.18 | 4.13  4.06, 4.19 | 4.15  4.08, 4.21 | 3.9×10^-9^ |  |
| P for interaction with study site |  |  |  |  |  |  | 0.09 |  |
| Women |  |  |  |  |  |  |  |  |
| Main multivariable model | +0.025 | 4.04  3.98, 4.10 | 4.10  4.04, 4.15 | 4.11  4.05, 4.16 | 4.12  4.07, 4.18 | 4.16  4.10, 4.21 | 3.4×10^-8^ |  |
| Men |  |  |  |  |  |  |  |  |
| Main multivariable model | +0.022 | 3.94  3.88, 4.00 | 3.96  3.90, 4.02 | 4.00  3.93, 4.06 | 4.00  3.94, 4.07 | 4.02  3.95, 4.08 | 1.0×10^-5^ |  |
| P for interaction with gender |  |  |  |  |  |  | 0.35 |  |
|  |  |  |  |  |  |  |  |  |
| BMI ≤25 |  |  |  |  |  |  |  |  |
| Main multivariable model | +0.015 | 3.99  3.91, 4.06 | 4.05  3.98. 4.12 | 4.04  3.97, 4.12 | 4.03  3.96, 4.10 | 4.08  4.01, 4.15 | 0.006 |  |
| BMI >25 |  |  |  |  |  |  |  |  |
| Main multivariable model | +0.026 | 3.99  3.94, 4.04 | 4.01  3.96, 4.06 | 4.06  4.00, 4.11 | 4.08  4.02, 4.13 | 4.08  4.03, 4.14 | 1.3×10^-9^ |  |
| P for interaction with BMI status |  |  |  |  |  |  | 0.43 |  |

^a^Adjusted for age, total energy intake, smoking and leisure time physical activity and education, and for gender and study site when applicable. General linear model.

*Supplementary Table 6a.* Shannon index in categories of the Swedish dietary guideline index (SweDGI) components in 9,101 women and men from SCAPIS Malmö and Uppsala

|  |  |  | Component score^b^ | | | | | |  |
| --- | --- | --- | --- | --- | --- | --- | --- | --- | --- |
| All | Beta^a^ | Beta^b^ | 0 | 1 | 2 | 3 | 4 | P-trend^a^ | P-trend^b^ |
| Fruits and berries | +0.014 | +0.0086 | 4.03  3.99, 4.07 | 4.06  4.02, 4.10 | 4.07  4.02, 4.11 | 4.06  4.02, 4.10 | 4.07  4.03, 4.11 | 8.1×10^-6^ | 0.014 |
| Vegetables | +0.025 | +0.013 | 4.02  3.98, 4.06 | 4.03  3.99, 4.07 | 4.08  4.04, 4.12 | 4.06  4.01, 4.10 | 4.08  4.03, 4.11 | 1.5×10^-14^ | 4.6×10^-4^ |
|  |  |  |  |  |  |  |  |  |  |
| Legumes | +0.010 | +0.00085 | 4.05  4.02, 4.09 | 4.08  4.04, 4.13 | 4.06  4.00, 4.12 | 4.03  3.96, 4.10 | 4.06  4.00, 4.11 | 0.037 | 0.86 |
|  |  |  |  |  |  |  |  |  |  |
| Nuts | +0.013 | +0.0043 | 4.05  4.01, 4.09 | 4.07  4.02, 4.11 | 4.10  4.05, 4.16 | 4.07  4.02, 4.11 | 4.03  3.98, 4.09 | 6.0×10^-3^ | 0.28 |
| Whole grain | +0.0024 | +0.0053 | 4.04  4.00, 4.09 | 4.04  4.00, 4.08 | 4.06  4.01, 4.10 | 4.06  4.02,4.11 | 4.07  4.02, 4.11 | 0.41 | 0.088 |
| Oil-dressing | +0.021 | +0.012 | 4.04  4.00, 4.08 | 4.07  4.03, 4.10 | 4.08  4.04, 4.12 | 4.06  4.01, 4.10 | 4.08  4.03, 4.14 | 1.5×10^-8^ | 3.0×10^-3^ |
| Fish | +0.016 | +0.013 | 4.00  3.95, 4.04 | 4.05  4.01, 4.09 | 4.07  4.03, 4.11 | 4.05  4.00, 4.09 | 4.08  4.04, 4.12 | 3.0×10^-7^ | 5.7×10^-5^ |
| High fat dairy | -0.0040 | -0.0067 | 4.06  4.01, 4.10 | 4.07  4.03,4.12 | 4.06  4.02, 4.11 | 4.06  4.02, 4.10 | 4.03  3.99, 4.07 | 0.27 | 0.066 |
|  |  |  |  |  |  |  |  |  |  |
| Meat | +0.0083 | +0.0042 | 4.04  3.99, 4.08 | 4.04  3.99, 4.09 | 4.07  4.02, 4.11 | 4.05  4.01, 4.09 | 4.06  4.02, 4.10 | 0.014 | 0.25 |
|  |  |  |  |  |  |  |  |  |  |
| Added sugar | +0.016 | +0.0078 | 4.03  3.98, 4.08 | 4.04  3.98, 4.09 | 4.04  3.99, 4.09 | 4.06  4.02, 4.11 | 4.06  4.02, 4.10 | 2.8×10^-5^ | 0.079 |
|  |  |  |  |  |  |  |  |  |  |
| Alcohol | -0.023 | -0.028 | 4.10  4.04, 4.16 | 4.10  4.05,4.15 | 4.11  4.76,4.15 | 4.08  4.04, 4.12 | 4.02  3.99, 4.06 | 1.2×10^-7^ | 4.1×10^-10^ |
|  |  |  |  |  |  |  |  |  |  |
| Salt | +0.0046 | +0.018 | 4.04  4.00, 4.09 | 4.03  3.99, 4.07 | 4.06  4.02, 4.10 | 4.09  4.04, 4.13 | 4.09  4.04, 4.15 | 0.29 | 1.1×10^-3^ |

^a^ Adjusted for age, sex, study site, total energy intake, smoking and leisure time physical activity and education.

^b^Mutual adjustment for all SweDGI food components.

*Supplementary Table 6b.* Shannon index in categories of the energy adjusted Swedish dietary guideline index (SweDGI) components in 9,101 women and men from SCAPIS Malmö and Uppsala

|  |  |  | Component score based on cut-points expressed as g/MJ ^b^ | | | | | |  |
| --- | --- | --- | --- | --- | --- | --- | --- | --- | --- |
| All | Beta^a^ | Beta^b^ | 0 | 1 | 2 | 3 | 4 | P-trend^a^ | P-trend^b^ |
| Fruits and berries | +0.012 | +0.0062 | 4.03  3.99, 4.08 | 4.05  4.01, 4.09 | 4.08  4.04, 4.12 | 4.08  4.03, 4.12 | 4.06  4.02, 4.10 | 6.4×10^-5^ | 0.052 |
| Vegetables | +0.025 | +0.016 | 4.00  3.95, 4.04 | 4.05  4.01, 4.09 | 4.06  4.01, 4.10 | 4.07  4.03, 4.11 | 4.08  4.04, 4.11 | 1.5×10^-14^ | 2.9×10^-5^ |
|  |  |  |  |  |  |  |  |  |  |
| Legumes | +0.0073 | +0.0010 | 4.06  4.02, 4.10 | 4.04  4.00, 4.09 | 4.08  4.02, 4.13 | 4.05  3.98, 4.11 | 4.07  4.02, 4.12 | 0.059 | 0.80 |
|  |  |  |  |  |  |  |  |  |  |
| Nuts | +0.014 | +0.0082 | 4.04  4.01, 4.08 | 4.07  4.03, 4.12 | 4.08  4.03, 4.12 | 4.09  4.04, 4.14 | 4.07  4.03, 4.12 | 4.2×10^-7^ | 6.4×10^-3^ |
| Whole grain | +0.0024 | +0.0071 | 4.05  4.00, 4.09 | 4.04  4.00, 4.08 | 4.06  4.02, 4.10 | 4.07  4.03,4.11 | 4.07  4.03, 4.12 | 0.41 | 0.025 |
| Oil-dressing | +0.016 | +0.0092 | 4.05  4.01, 4.08 | 4.06  4.02, 4.11 | 4.08  4.023 4.13 | 4.07  4.02, 4.12 | 4.08  4.04, 4.12 | 3.3×10^-8^ | 3.2×10^-3^ |
| Fish | +0.021 | +0.017 | 3.97  3.92, 4.02 | 4.04  4.00, 4.09 | 4.05  4.01, 4.09 | 4.04  4.00, 4.09 | 4.08  4.04, 4.12 | 4.9×10^-10^ | 1.6×10^-6^ |
| High fat dairy | -0.0036 | -0.0060 | 4.06  4.02, 4.10 | 4.07  4.03,4.11 | 4.06  4.02, 4.10 | 4.04  3.99, 4.08 | 4.04  4.00, 4.09 | 0.26 | 0.063 |
|  |  |  |  |  |  |  |  |  |  |
| Meat | +0.0017 | -0.0024 | 4.06  4.02, 4.10 | 4.07  4.02, 4.12 | 4.06  4.02, 4.10 | 4.05  4.00, 4.10 | 4.05  4.01, 4.09 | 0.54 | 0.44 |
|  |  |  |  |  |  |  |  |  |  |
| Added sugar | +0.016 | +0.0062 | 4.04  3.99, 4.09 | 4.04  3.98, 4.10 | 4.04  4.00, 4.09 | 4.07  4.02, 4.11 | 4.06  4.02, 4.10 | 2.8×10^-5^ | 0.16 |
|  |  |  |  |  |  |  |  |  |  |
| Alcohol | -0.023 | -0.024 | 4.09  4.04, 4.15 | 4.09  4.04,4.15 | 4.11  4.06,4.15 | 4.08  4.04, 4.12 | 4.03  3.99, 4.07 | 1.2×10^-7^ | 7.8×10^-8^ |
| Salt | +0.0046 | +0.021 | 4.07  4.02, 4.11 | 4.06  4.2, 4.10 | 4.08  4.04, 4.12 | 4.12  4.07, 4.16 | 4.13  4.07, 4.19 | 0.29 | 1.1×10^-4^ |
|  |  |  |  |  |  |  |  |  |  |

^a^ Adjusted for age, sex, study site, total energy intake, smoking and leisure time physical activity and education.

^b^Mutual adjustment for all SweDGI food components.

*Supplementary Table 7.* Odds ratios with confidence intervals for prevalence of the metabolic syndrome according to adherence to the Swedish dietary guideline index (SweDGI) in 10,396 women and men from SCAPIS Malmö and Uppsala

|  |  | Category of SweDGI | | | | | |  |
| --- | --- | --- | --- | --- | --- | --- | --- | --- |
| All | Beta^a^ | 1  ≤ 18 | 2  19-22 | 3  23-25 | 4  26-29 | 5  ≥ 30 | P-trend | P-trend  energy-adjusted SweDGI |
| Basic model^b^ | -0.21 | 1.00 | 0.76  0.67, 0.86 | 0.63  0.55, 0.72 | 0.52  0.45, 0.60 | 0.42  0.36, 0.49 | 1.3×10^-36^ | 1.1×10^-35^ |
| Main multivariable model^c^ | -0.14 | 1.00 | 0.85 | 0.77 | 0.68 | 0.59 | 6.4×10^-13^ | 1.0×10^-12^ |
|  |  |  | 0.75, 0.97 | 0.67, 0.88 | 0.59, 0.78 | 0.51, 0.69 |  |  |
| Multivariable model with BMI | -0.08 | 1.00 | 0.87 | 0.79 | 0.76 | 0.71 | 1.2×10^-5^ | 4.6×10^-5^ |
|  |  |  | 0.76, 0.99 | 0.68, 0.92 | 0.65, 0.88 | 0.60, 0.84 |  |  |
| Multivariable model + Shannon^d^ | -0.12 | 1.00 | 0.86  0.75, 0.97 | 0.79  0.68, 0.90 | 0.69  0.60, 0.80 | 0.61  0.52, 0.72 | 3.3×10^-11^ | 4.7×10^-11^ |
| Multivariable model + Fibre | -0.14 | 1.00 | 0.84  0.73, 0.97 | 0.75  0.64, 0.89 | 0.71  0.59, 0.84 | 0.64  0.52, 0.79 | 2.1×10^-9^ | 3.8×10^-9^ |
| Multivariable model +BMI + Shannon^d^ | -0.08 | 1.00 | 0.87  0.76, 1.00 | 0.80  0.69, 0.93 | 0.77  0.66, 0.90 | 0.72  0.61, 0.86 | 4.3×10^-5^ | 1.5×10^-4^ |
| Multivariable model +BMI + fibre | -0.10 | 1.00 | 0.85  0.73, 0.98 | 0.76  0.65, 0.90 | 0.72  0.60, 0.86 | 0.66  0.54, 0.82 | 6.1×10^-5^ | 3.2×10^-4^ |

^a^Beta indicates mean difference per category of SweDGI.

^b^Adjusted for age, sex, study site and total energy intake.

^c^Adjusted for age, sex, study site, total energy intake, smoking and leisure time physical activity and education.

^d^Missing on Shannon index was replaced by mean Shannon index in 1295 individuals.

*Supplementary Table 8.* Odds ratios with confidence intervals for prevalence of the metabolic syndrome according to adherence to the Swedish dietary guideline index (SweDGI) in site, gender and BMI stratified analyses among 5,726 Malmö-participants and 4,670 Uppsala-participants, among 5,516 women and 4,880 men, and among 3,716 participants with BMI ≤25 and 6680 participants with BMI >25 from SCAPIS

|  |  | Category of SweDGI | | | | | |  |
| --- | --- | --- | --- | --- | --- | --- | --- | --- |
| All | Beta^a^ | 1  ≤ 18 | 2  19-22 | 3  23-25 | 4  26-29 | 5  ≥ 30 | P-trend^b^ |  |
| Malmö |  |  |  |  |  |  |  |  |
| Main multivariable model | -0.11 | 1.00 | 0.83 | 0.81 | 0.68 | 0.65 | 7.7×10^-6^ |  |
|  |  |  | 0.70, 0.99 | 0.67, 0.97 | 0.56, 0.83 | 0.52, 0.80 |  |  |
| Uppsala |  |  |  |  |  |  |  |  |
| Main multivariable model | -0.15 | 1.00 | 0.88  0.73, 1.07 | 0.73  0.59, 0.90 | 0.68  0.55, 0.84 | 0.54  0.42, 0.68 | 1.9×10^-8^ |  |
| P for interaction with study site |  |  |  |  |  |  | 0.07 |  |
| Women |  |  |  |  |  |  |  |  |
| Main multivariable model | -0.13 | 1.00 | 0.85 | 0.71 | 0.70 | 0.55 | 3.7×10^-8^ |  |
|  |  |  | 0.69, 1.06 | 0.57, 0.89 | 0.57, 0.86 | 0.44, 0.69 |  |  |
| Men |  |  |  |  |  |  |  |  |
| Main multivariable model | -0.11 | 1.00 | 0.83  0.71, 0.97 | 0.82  0.68, 0.99 | 0.70  0.58, 0.85 | 0.62  0.48, 0.81 | 8.2×10^-6^ |  |
| P for interaction with gender  BMI ≤25 |  |  |  |  |  |  | 0.70 |  |
| Main multivariable model | -0.22 | 1.00 | 0.96 | 0.59 | 0.62 | 0.42 | 2.4×10^-6^ |  |
|  |  |  | 0.68, 1.36 | 0.40, 0.88 | 0.42, 0.90 | 0.28, 0.63 |  |  |
|  |  |  |  |  |  |  |  |  |
| BMI>25 |  |  |  |  |  |  |  |  |
| Main multivariable model | -0.07 | 1.00 | 0.84  0.73, 0.98 | 0.80  0.68, 0.93 | 0.74  0.63, 0.87 | 0.75  0.63, 0.90 | 2.6×10^-4^ |  |
| P for interaction with gender |  |  |  |  |  |  | 0.046 |  |

^a^Beta indicates mean difference per category of SweDGI.

^b^Adjusted for age, sex, study site, total energy intake, smoking and leisure time physical activity and education when applicable.

*Supplementary Table 9.* Adherence to the SWEDGI and odds ratios of having components of the metabolic syndrome

|  |  |  | Category of SweDGI^c^ | | | | | |  |
| --- | --- | --- | --- | --- | --- | --- | --- | --- | --- |
| Components of the metabolic syndrome | Beta^ab^ | Beta^ac^ | 1  ≤ 18 | 2  19-22 | 3  23-25 | 4  26-29 | 5  ≥ 30 | P-trend^b^ | P-trend^c^ |
| Waist circumference, high  (women: ≥ 80, men: ≥ 94cm,) | -0.18 | -0.16 | 1.00 | 0.75  0.64, 0.88 | 0.74  0.62, 0.88 | 0.61  0.52, 0.72 | 0.50  0.42, 0.60 | 3.6×10^-22^ | 9.2×10^-16^ |
| Blood pressure, high  (SBP ≥ 130/DBP ≥ 85 mmHg) | -0.11 | -0.08 | 1.00 | 0.87  0.77, 0.99 | 0.83  0.72, 0.95 | 0.77  0.67, 0.88 | 0.71  0.62, 0.82 | 4.5×10^-11^ | 2.1×10^-6^ |
| Glucose, high (≥ 5.6 mmol/L) | -0.06 | -0.04 | 1.00 | 0.92  0.82, 1.05 | 0.97  0.85, 1.10 | 0.87  0.76, 0.99 | 0.85  0.74, 0.98 | 3.2×10^-4^ | 0.019 |
|  |  |  |  |  |  |  |  |  |  |
| Triglycerides, high  (≥ 1.7 mmol/L) | -0.10 | -0.06 | 1.00 | 0.92  0.79, 1.06 | 0.89  0.76, 1.04 | 0.84  0.72, 0.99 | 0.77  0.65, 0.92 | 1.5×10^-7^ | 3.8×10^-3^ |
|  |  |  |  |  |  |  |  |  |  |
| HDL-cholesterol, low  (women: ≤ 1.29 mmol/L, men: ≤ 1.03) | -0.06 | +0.01 | 1.00 | 1.10  0.90, 1.35 | 1.08  0.86, 1.34 | 1.06  0.85, 1.33 | 1.04  0.82, 1.33 | 0.029 | 0.83 |

HDL-cholesterol, high-density lipoprotein cholesterol.

^a^Beta indicates mean difference per category of SweDGI.

^b^ Main multivariable model, adjusted for age, sex, study site, total energy intake, smoking and leisure time physical activity and education.

^c^ Main multivariable model + mutual adjustment for all components of the metabolic syndrome.

*Supplementary Table 10a.* Scores of the Swedish dietary guideline index (SweDGI) components and odds ratios of prevalent metabolic syndrome

|  |  |  | Score for SweDGI components | | | | |  |  |
| --- | --- | --- | --- | --- | --- | --- | --- | --- | --- |
| Components of the SweDGI | Beta^a^ | Beta^b^ | 0 | 1 | 2 | 3 | 4 | P-trend^a^ | P-trend^b^ |
| Fruits and berries | -0.05 | -0.002 | 1.00 | 0.92  0.82, 1.04 | 0.77  0.68, 0.88 | 0.87  0.75, 1.01 | 0.80  0.69, 0.92 | 2.4×10^-3^ | 0.91 |
|  |  |  |  |  |  |  |  |  |  |
| Vegetables | -0.06 | -0.04 | 1.00 | 0.96  0.83, 1.10 | 0.90  0.78, 1.05 | 0.93  0.79, 1.11 | 0.84  0.72, 0.99 | 7.8×10^-5^ | 0.04 |
|  |  |  |  |  |  |  |  |  |  |
| Legumes | -0.07 | -0.02 | 1.00 | 0.93  0.79, 1.10 | 0.86  0.67, 1.11 | 0.92  0.66, 1.27 | 0.71  0.55, 0.93 | 8.9×10^-3^ | 0.38 |
|  |  |  |  |  |  |  |  |  |  |
| Nuts | -0.12 | -0.09 | 1.00 | 0.86  0.75, 1.00 | 0.72  0.57, 0.91 | 0.73  0.62, 0.85 | 0.86  0.65, 1.13 | 9.5×10^-10^ | 2.6×10^-5^ |
|  |  |  |  |  |  |  |  |  |  |
| Whole grain | -0.06 | -0.05 | 1.00 | 0.92  0.80, 1.06 | 0.90  0.78, 1.03 | 0.83  0.71, 0.96 | 0.81  0.70, 0.93 | 1.4×10^-4^ | 1.4×10^-3^ |
|  |  |  |  |  |  |  |  |  |  |
| Fish | +0.01 | +0.01 | 1.00 | 1.01  0.87, 1.17 | 1.15  0.98, 1.34 | 1.12  0.95, 1.33 | 1.06  0.91, 1.22 | 0.59 | 0.54 |
|  |  |  |  |  |  |  |  |  |  |
| Oil-dressing | -0.06 | -0.02 | 1.00 | 0.94  0.84, 1.04 | 1.00  0.88, 1.14 | 0.73  0.61, 0.87 | 0.78  0.62, 0.98 | 6.2×10^-3^ | 0.29 |
|  |  |  |  |  |  |  |  |  |  |
| High fat dairy | +0.05 | +0.06 | 1.00 | 1.10  0.93, 1.30 | 1.06  0.91, 1.23 | 1.20  1.03, 1.40 | 1.26  1.06, 1.49 | 1.2×10^-3^ | 2.9×10^-3^ |
|  |  |  |  |  |  |  |  |  |  |
| Red meat | -0.09 | -0.05 | 1.00 | 0.91  0.74, 1.12 | 0.74  0.61, 0.89 | 0.80  0.67, 0.96 | 0.78  0.66, 0.91 | 1.6×10^-7^ | 4.7×10^-3^ |
|  |  |  |  |  |  |  |  |  |  |
| Added sugar | -0.08 | -0.05 | 1.00 | 0.76  0.60, 1.01 | 0.77  0.62, 0.97 | 0.67  0.54, 0.83 | 0.75  0.62, 0.92 | 9.3×10^-6^ | 0.028 |
|  |  |  |  |  |  |  |  |  |  |
| Alcohol | -0.06 | -0.03 | 1.00 | 0.87  0.67, 1.14 | 0.64  0.50, 0.80 | 0.59  0.48, 0.74 | 0.68  0.55, 0.84 | 3.6×10^-3^ | 0.15 |
|  |  |  |  |  |  |  |  |  |  |
| Salt | -0.05 | -0.05 | 1.00 | 1.00  0.85, 1.18 | 0.89  0.75, 1.06 | 0.87  0.71, 1.06 | 0.90  0.68, 1.18 | 0.016 | 0.064 |

^a^ Multivariable model: Adjusted for age, sex, study site, total energy intake, smoking and leisure time physical activity and education.

^b^ Multivariable model + mutual adjustment for all food components.

*Supplementary Table 10b.* Scores of the energy adjusted Swedish dietary guideline index (SweDGI) components and odds ratios of prevalent metabolic syndrome

|  |  | SweDGI component scores based on cut-points expressed in g/MJ (mutual adjusted) | | | | |  |  |
| --- | --- | --- | --- | --- | --- | --- | --- | --- |
| Components of the SweDGI | Beta^b^ | 0 | 1 | 2 | 3 | 4 | P-trend^a^ | P-trend^b^ |
| Fruits and berries | +0.01 | 1.00 | 1.04  0.90, 1.19 | 0.94  0.81, 1.09 | 0.93  0.79, 1.10 | 1.05  0.91, 1.21 | 0.027 | 0.50 |
|  |  |  |  |  |  |  |  |  |
| Vegetables | -0.04 | 1.00 | 0.99  0.83, 1.17 | 0.91  0.76, 1.09 | 0.83  0.69, 1.01 | 0.86  0.72, 1.02 | 2.4×10^-4^ | 0.029 |
|  |  |  |  |  |  |  |  |  |
| Legumes | -0.02 | 1.00 | 1.02  0.88, 1.17 | 0.88  0.70, 1.12 | 1.08  0.81, 1.44 | 0.90  0.74, 1.11 | 0.011 | 0.38 |
|  |  |  |  |  |  |  |  |  |
| Nuts | -0.07 | 1.00 | 0.96  0.85, 1.08 | 0.93  0.79, 1.11 | 0.75  0.62, 0.91 | 0.74  0.65, 0.85 | 2.6×10^-10^ | 7.5×10^-6^ |
|  |  |  |  |  |  |  |  |  |
| Whole grain | -0.05 | 1.00 | 0.91  0.79, 1.05 | 0.92  0.80, 1.05 | 0.85  0.73, 0.99 | 0.84  0.74, 0.97 | 1.4×10^-4^ | 1.8×10^-3^ |
|  |  |  |  |  |  |  |  |  |
| Fish | +0.01 | 1.00 | 0.91  0.74, 1.12 | 0.97  0.79, 1.18 | 0.99  0.81, 1.21 | 1.01  0.84,1.22 | 0.45 | 0.57 |
|  |  |  |  |  |  |  |  |  |
| Oil-dressing | -0.01 | 1.00 | 0.95  0.84, 1.08 | 0.99  0.85, 1.16 | 1.04  0.88, 1.24 | 0.91  0.78, 1.05 | 0.039 | 0.58 |
|  |  |  |  |  |  |  |  |  |
| High fat dairy | +0.04 | 1.00 | 1.02  0.90, 1.15 | 1.07  0.95, 1.21 | 1.02  0.89, 1.18 | 1.18  1.00, 1.38 | 0.026 | 0.03 |
|  |  |  |  |  |  |  |  |  |
| Meat | -0.05 | 1.00 | 1.00  0.86, 1.17 | 0.90  0.77, 1.05 | 0.85  0.73, 1.00 | 0.82  0.72, 0.93 | 2.4×10^-9^ | 3.3×10^-3^ |
|  |  |  |  |  |  |  |  |  |
| Added sugar | -0.05 | 1.00 | 0.77  0.58, 1.01 | 0.79  0.63, 0.99 | 0.68  0.55, 0.84 | 0.75  0.62, 0.92 | 9.3×10^-6^ | 0.015 |
|  |  |  |  |  |  |  |  |  |
| Alcohol | -0.04 | 1.00 | 0.89  0.68, 1.16 | 0.67  0.53, 0.84 | 0.64  0.52, 0.80 | 0.76  0.61, 0.94 | 3.6×10^-3^ | 0.11 |
|  |  |  |  |  |  |  |  |  |
| Salt | -0.06 | 1.00 | 1.00  0.85, 1.18 | 0.89  0.75, 1.06 | 0.87  0.71, 1.07 | 0.91  0.69, 1.20 | 0.016 | 0.0498 |

^a^ Multivariable model: Adjusted for age, sex, study site, total energy intake, smoking and leisure time physical activity and education.

^b^Multivariable model + mutual adjustment for all food components

*Supplementary Table 11.* Odds ratios with confidence intervals for prevalence of the metabolic syndrome according to Quintile of Shannon index in 9,101 women and men from SCAPIS Malmö and Uppsala

|  |  | Quintile of Shannon index | | | | | |  |
| --- | --- | --- | --- | --- | --- | --- | --- | --- |
| All | Beta^a^ | 1 | 2 | 3 | 4 | 5 | P-trend |  |
| Basic model^b^ | -0.22 | 1.00 | 0.84  0.74, 0.97 | 0.63  0.55, 0.72 | 0.50  0.44, 0.58 | 0.42  0.37, 0.49 | 2.9×10^-40^ |  |
|  |  |  |  |  |  |  |  |  |
| Main multivariable model^c^ | -0.17 | 1.00 | 0.90 | 0.70 | 0.58 | 0.53 | 1.3×10^-23^ |  |
|  |  |  | 0.78, 1.04 | 0.60, 0.81 | 0.50, 0.67 | 0.45, 0.61 |  |  |
|  |  |  |  |  |  |  |  |  |
| Multivariable model with BMI | -0.10 | 1.00 | 0.97 | 0.83 | 0.72 | 0.75 | 5.7×10^-8^ |  |
|  |  |  | 0.84, 1.13 | 0.71, 0.96 | 0.61, 0.84 | 0.63, 0.88 |  |  |
|  |  |  |  |  |  |  |  |  |
| Multivariable model + SweDGI^d^ | -0.17 | 1.00 | 0.91  0.79, 1.04 | 0.71  0.62, 0.82 | 0.60  0.52, 0.69 | 0.57  0.49, 0.66 | 7.7×10^-22^ |  |
| Multivariable model+BMI+SweDGI | -0.10 | 1.00 | 0.97  0.84, 1.13 | 0.83  0.71, 0.97 | 0.72  0.62, 0.85 | 0.76  0.64 0.89 | 1.9×10^-7^ |  |

^a^Beta indicates mean difference per category of Shannon index.

^b^Adjusted for age, sex and study site.

^c^Adjusted for age, sex, study site, smoking and leisure time physical activity and education.

^d^Swedish dietary guideline index.

*Supplementary Table 12.* Shannon index and odds ratios of having components of metabolic syndrome

|  |  | Quintile of Shannon index^b^ | | | | | |  |
| --- | --- | --- | --- | --- | --- | --- | --- | --- |
| Components of the metabolic syndrome | Beta^ab^ | 1 | 2 | 3 | 4 | 5 | P-trend^b^ | P-trend^c^ |
| Waist circumference, high  (women: ≥ 80, men: ≥ 94cm,) | -0.14 | 1.00 | 0.84  0.71, 1.00 | 0.69  0.58, 0.81 | 0.69  0.58, 0.81 | 0.55  0.46, 0.64 | 9.8×10^-15^ | 4.0×10^-7^ |
|  |  |  |  |  |  |  |  |  |
| Blood pressure, high  (SBP ≥ 130/DBP ≥ 85 mmHg) | -0.07 | 1.00 | 0.85  0.74, 0.97 | 0.78  0.67, 0.89 | 0.72  0.63, 0.83 | 0.77  0.67, 0.89 | 2.3×10^-5^ | 0.08 |
| Glucose, high (≥ 5.6 mmol/L) | -0.08 | 1.00 | 0.97  0.85, 1.10 | 0.88  0.67, 1.01 | 0.79  0.69, 0.90 | 0.72  0.63, 0.83 | 5.6×10^-8^ | 1.0×10^-5^ |
|  |  |  |  |  |  |  |  |  |
| Triglycerides, high  (≥ 1.7 mmol/L) | -0.20 | 1.00 | 0.84  0.72, 0.97 | 0.62  0.74, 0.73 | 0.55  0.47, 0.64 | 0.45  0.38, 0.54 | 6.4×10^-27^ | 5.6×10^-16^ |
|  |  |  |  |  |  |  |  |  |
| HDL-cholesterol, low  (women: ≤ 1.29 mmol/L, men: ≤ 1.03) | -0.17 | 1.00 | 0.95  0.87, 1.15 | 0.91  0.49, 0.75 | 0.71  0.58, 0.88 | 0.50  0.39, 0.63 | 6.4×10^-11^ | 0.02 |

HDL-cholesterol, High-density lipoprotein cholesterol

^a^ Beta indicates mean difference per quintile of Shannon index.

^b^ Main multivariable model: adjusted for age, sex, study site, smoking and leisure time physical activity and education.

^c^ Main multivariable model + mutual adjustment for all components of the metabolic syndrome.

*Supplementary Table 13.* Odds ratios ^a^ with confidence intervals for prevalence of the metabolic syndrome according to tertile of Shannon index

in strata of the SweDGI in 9,101 women and men from SCAPIS Malmö and Uppsala

| SweDGI  Category  (Tertile) | Tertile of Shannon index | | | P  for trend^b^ |
| --- | --- | --- | --- | --- |
|  | Low | Medium | High |  |
|  |  |  |  |  |
| Low  (6-21) | 1.00 | 0.72  0.60, 0.85 | 0.56  0.47, 0.68 | 1.2×10^-9^ |
| Medium (22-27) | 1.00 | 0.66  0.54, 0.80 | 0.54  0.44, 0.66 | 2.2×10^-9^ |
| High  (28-45) | 1.00 | 0.75  0.60, 0.95 | 0.59  0.46, 0.74 | 1.2×10^-5^ |
|  |  |  |  |  |
| P for interaction^c^ |  |  |  | 0.82 |

^a^ Adjusted for age, sex, study site, total energy intake, smoking and leisure time physical activity and education.

^b^ P for trend across tertiles of Shannon index in tertiles of adherence to the high-fat dairy guideline.

^c^ P for interaction treating the categories as continuous variables.

*Supplementary Table 14.* Odds ratios ^a^ with confidence intervals for prevalence of the metabolic syndrome according to level of the SweDGI

in strata of Shannon index in 9,101 women and men from SCAPIS Malmö and Uppsala

|  |  | | | |
| --- | --- | --- | --- | --- |
| SweDGI  Category  (Tertile) | Tertile of Shannon index | | |  |
|  | Low | Medium | High |  |
|  |  |  |  |  |
| Poor  (1) | 1.00 | 1.00 | 1.00 |  |
| Medium  (2) | 0.91  0.76, 1.09 | 0.85  0.70, 1.04 | 0.91  0.73, 1.13 |  |
| Good  (3) | 0.69  0.56, 0.86 | 0.76  0.61, 0.96 | 0.80  0.62, 1.02 |  |
| P_trend_^b^ | 0.002 | 0.018 | 0.071 |  |
| P_interaction_^c^ |  |  |  | 0.82 |

^a^ Adjusted for age, sex, study site, total energy intake, smoking and leisure time physical activity and education.

^b^ P for trend across tertiles of adherence to the high fat dairy recommendation in tertiles of Shannon index.

^c^ P for interaction treating the categories as continuous variables.

*Supplementary Table 15.* Odds ratios ^a^ with confidence intervals for prevalence of the metabolic syndrome according to combinations of tertile of Shannon index and three levels of the Swedish dietary guideline index (SweDGI) in 9,101 women and men from SCAPIS Malmö and Uppsala

| SweDGI  Category  (Score) | Tertile of Shannon index | | | P  for trend^c^ |
| --- | --- | --- | --- | --- |
|  | Low | Medium | High |  |
|  |  |  |  |  |
| Low  (6-21) | 1.00^b^ | 0.72  0.60, 0.85 | 0.57  0.47, 0.68 | 1.2×10^-9^ |
| Medium (22-27) | 0.93  0.78, 1.11 | 0.61  0.51, 0.73 | 0.50  0.42, 0.61 | 2.2×10^-9^ |
| High  (28-45) | 0.72  0.58, 0.89 | 0.55  0.44, 0.67 | 0.43  0.35, 0.52 | 1.2×10^-5^ |
| P_trend_^d^ | 0.002 | 0.018 | 0.071 |  |
| P_interaction_^e^ |  |  |  | 0.82 |

^a^ Adjusted for age, sex, study site, total energy intake, smoking and leisure time physical activity and education.

^b^ Reference in joint effect model.

^c^ P for trend across tertiles of Shannon index in categories of SweDGI.

^d^ P for trend across categories of SweDGI in tertiles of Shannon index.

^e^ P for interaction treating the categories as continuous variables. In parenthesis, P for interaction between continuous variables of Shannon index and the SweDGI.

*Supplementary Table 16.* Interactions between food group levels and tertile of Shannon index in 9,101 women and men from SCAPIS Malmö and Uppsala

|  | Fruits and berries | | Vegetables | | Legumes | | Nuts and seeds | | Whole grain | | Fish and shellfish | | Oil-dressing | | High-fat dairy | | Red meat | | Added sugar | | Alcohol | | Salt |  |
| --- | --- | --- | --- | --- | --- | --- | --- | --- | --- | --- | --- | --- | --- | --- | --- | --- | --- | --- | --- | --- | --- | --- | --- | --- |
|  |  | |  | |  | |  | |  | |  | |  | |  | |  | |  | |  | |  |  |
| P for interaction^a^ | 0.40 | | 0.92 | | 0.14 | | 0.92 | | 0.36 | | 0.44 | | 0.37 | | 0.04 | | 0.37 | | 0.72 | | 0.09 | | 0.78 |  |
|  |  |  | |  | |  | |  | |  | |  | |  | |  | |  | |  | |  | |  |

^a^Adjusted for age, sex, study site, total energy intake, smoking and leisure time physical activity and education. P for interaction treating tertiles of Shannon index and foods as continuous variables.

*Supplementary Table 17.* Odds ratios^a^ with confidence intervals for prevalence of the metabolic syndrome according to level of adherence to the high fat dairy recommendation in strata of Shannon in 9,101 women and men from SCAPIS Malmö and Uppsala

|  |  | | | |
| --- | --- | --- | --- | --- |
| Adherence to  high-fat dairy guideline  (Tertile) | Tertile of Shannon index | | |  |
|  | Low | Medium | High |  |
|  |  |  |  |  |
| Poor  (1) | 1.00 | 1.00  0.61, 0.86 | 1.00  0.48, 0.70 |  |
| Medium  (2) | 1.20  0.99, 1.47 | 1.16  0.94, 1.45 | 1.16  0.94, 1.44 |  |
| Good  (3) | 1.06  0.87, 1.30 | 1.29  1.01, 1.56 | 1.29  1.04, 1.61 |  |
| P_trend_^b^ | 0.65 | 0.02 | 0.01 |  |
| P_interaction_^c^ |  |  |  | 0.04 |

^a^ Adjusted for age, sex, study site, total energy intake, smoking and leisure time physical activity and education

^b^ P for trend across tertiles of adherence to the high fat dairy recommendation in tertiles of Shannon index

^c^ P for interaction treating the categories as continuous variables.

*Supplementary Table 18.* Odds ratios ^a^ with confidence intervals for prevalence of the metabolic syndrome according to tertile of Shannon index in strata of adherence to the high fat dairy recommendation in 9,101 women and men from SCAPIS Malmö and Uppsala

|  |  | | | |
| --- | --- | --- | --- | --- |
| Adherence to  high-fat dairy guideline  (Tertile) | Tertile of Shannon index | | | P  for trend^b^ |
|  | Low | Medium | High |  |
|  |  |  |  |  |
| Poor  (1) | 1.00 | 0.68  0.55, 0.82 | 0.52  0.42, 0.64 | 8.3×10^-10^ |
| Medium  (2) | 1.00 | 0.63  0.52, 0.77 | 0.51  0.42, 0.63 | 5.6×10^-11^ |
| Good  (3) | 1.00 | 0.79  0.65, 0.99 | 0.64  0.52, 0.78 | 1.4×10^-5^ |
|  |  |  |  |  |
| P_interaction_^c^ |  |  |  | 0.04 |

^a^ Adjusted for age, sex, study site, total energy intake, smoking and leisure time physical activity and education.

^b^ P for trend across tertiles of Shannon index in tertiles of adherence to the high-fat dairy guideline.

^c^ P for interaction treating the categories as continuous variables.
